# Polyploidisation and Geographic Differentiation Drive Diversification in a European High Mountain Plant Group (*Doronicum clusii* Aggregate, Asteraceae)

**DOI:** 10.1371/journal.pone.0118197

**Published:** 2015-03-06

**Authors:** Clemens Pachschwöll, Pedro Escobar García, Manuela Winkler, Gerald M. Schneeweiss, Peter Schönswetter

**Affiliations:** 1 Department of Botany and Biodiversity Research, University of Vienna, Rennweg 14, A-1030 Vienna, Austria; 2 Department of Botany, Natural History Museum, Burgring 7, A-1010 Vienna, Austria; 3 GLORIA co-ordination, University of Natural Resources and Life Sciences Vienna, Center for Global Change and Sustainability & Austrian Academy of Sciences, Institute for Interdisciplinary Mountain Research, Silbergasse 30, A-1190 Vienna, Austria; 4 Institute of Botany, University of Innsbruck, Sternwartestrasse 15, A-6020 Innsbruck, Austria; University of Gottingen, GERMANY

## Abstract

Range shifts (especially during the Pleistocene), polyploidisation and hybridization are major factors affecting high-mountain biodiversity. A good system to study their role in the European high mountains is the *Doronicum clusii* aggregate (Asteraceae), whose four taxa (*D. clusii* s.s., *D. stiriacum*, *D. glaciale* subsp. *glaciale* and *D. glaciale* subsp. *calcareum*) are differentiated geographically, ecologically (basiphilous versus silicicolous) and/or via their ploidy levels (diploid versus tetraploid). Here, we use DNA sequences (three plastid and one nuclear spacer) and AFLP fingerprinting data generated for 58 populations to infer phylogenetic relationships, origin of polyploids—whose ploidy level was confirmed by chromosomally calibrated DNA ploidy level estimates—and phylogeographic history. Taxonomic conclusions were informed, among others, by a Gaussian clustering method for species delimitation using dominant multilocus data. Based on molecular data we identified three lineages: (i) silicicolous diploid *D. clusii* s.s. in the Alps, (ii) silicicolous tetraploid *D. stiriacum* in the eastern Alps (outside the range of *D. clusii* s.s.) and the Carpathians and (iii) the basiphilous diploids *D. glaciale* subsp. glaciale (eastern Alps) and *D. glaciale* subsp. *calcareum* (northeastern Alps); each taxon was identified as distinct by the Gaussian clustering, but the separation of *D. glaciale* subsp. *calcareum* and *D. glaciale* subsp. *glaciale* was not stable, supporting their taxonomic treatment as subspecies. Carpathian and Alpine populations of *D. stiriacum* were genetically differentiated suggesting phases of vicariance, probably during the Pleistocene. The origin (autopolyploid versus allopolyploid) of *D. stiriacum* remained unclear. *Doronicum glaciale* subsp. *calcareum* was genetically and morphologically weakly separated from *D. glaciale* subsp. *glaciale* but exhibited significantly higher genetic diversity and rarity. This suggests that the more widespread *D. glaciale* subsp. *glaciale* originated from *D. glaciale* subsp. *calcareum*, which is restricted to a prominent Pleistocene refugium previously identified in other alpine plant species.

## Introduction

Biota of temperate European mountain ranges such as the European Alps were strongly influenced by Quaternary climatic oscillations causing range shifts and extirpations [1–3]. Intersecting paleo-environmental (e.g., maximum extent of glaciers and extrapolated position of the snow line) with genetic data (patterns of genetic diversity and rarity, geographical distribution of genetically delimited groups), it was shown that during Pleistocene glaciations many alpine species were forced into only locally glaciated mountain ranges at or close to the periphery of the Alps [4–6]. Major peripheral refugia were situated in the southwestern and the southern Alps; for silicicolous and calcicolous species, additional refugia were located in the eastern-most and in the northeastern-most Alps, respectively. Refugia on nunataks in the strongly glaciated interior of the Alps were suggested for only a few species [7,8].

During phases of warmer climate, lowland populations of alpine species got extinct while ranges expanded from the refugia towards interior areas of the Alps [9,10]. In many species phases of isolation were prolonged by incomplete range filling (temperate trees [11]; alpine plants [12]) resulting in disjunct distributions [13–17], but even in species with contiguous distribution areas vicariance may still be evident in strong phylogeographic breaks and the presence of hybrid zones [18–23].

Quaternary climatic oscillations did, however, not only reshuffle distribution ranges but also fostered lineage diversification and speciation [24,25]. Ecogeographic isolation, such as edaphic specialization, and polyploidisation are among the most important mechanisms driving the diversification of angiosperms in general [26–29], and that of cold-adapted species in particular [30–37]. This is supported by the good congruence of refugial patterns with centres of endemism and species richness [38–42] or co-occurrence, partly over wider geographic scales, of intraspecific genetic groups [43–46].

A good system to study the role of range shifts, polyploidisation and hybridization for diversification of mountain biota is the Eurasian genus *Doronicum* (Asteraceae—Senecioneae). A sound hypothesis on relationships within *Doronicum* is available from revisionary taxonomic and molecular phylogenetic work [47–50]. In this genus polyploidy [50] and hybridization [51,52] are common phenomena and may be at least partly responsible for high morphological variation and delimitation problems [50,53,54]. Sexual reproduction and outcrossing was experimentally proven for the diploid species *D. austriacum* Jacq. and *D. grandiflorum* Lam. s.s. as well as for the tetraploid species *D. stiriacum* (Vill.) Dalla Torre (as *D. clusii* (All.) Tausch) [55,56]. In *Doronicum*, the cypselae possess long pappus bristles and are likely wind-dispersed [50,57].

**Fig 1:**
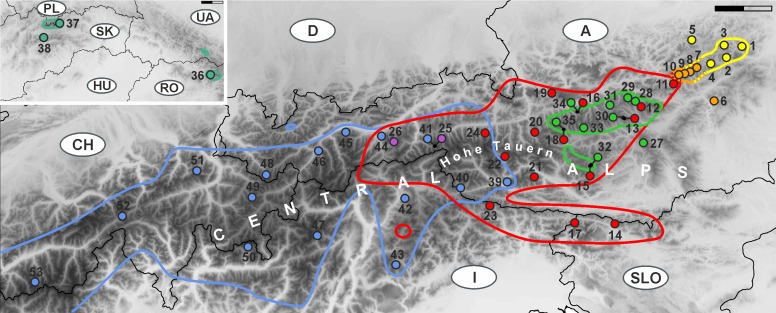
Investigated populations of the *Doronicum clusii* aggregate in the Alps and Carpathians. Population numbers are as in Table 2; taxonomic entities are colour-coded: *D. glaciale* subsp. *calcareum* (yellow), *D. glaciale* subsp. *glaciale* (red) and their morphological intermediates (orange); *D. stiriacum* (green); *D. clusii* s.s. (blue); hybrids between *D. clusii* s.s. and *D. glaciale* subsp. *glaciale* (*D. × bauhini*; lavender). The insert shows Carpathian populations of *D. stiriacum* (turquoise); for graphical reasons the insert is in the upper left despite the Carpathians being east of the Alps. The coloured outlines are distribution ranges summarized according to various literature sources, herbarium specimens and personal observations. Scale bars: 50 km.

The monophyletic *D. clusii* aggregate comprises four geographically, ecologically and/or karyologically differentiated subalpine to subnival forbs restricted to the European mountain systems of the Alps and the Carpathians [49,57,58,59]. They possess yellow, showy flower heads that are visited and pollinated by various Diptera, Hymenoptera and Lepidoptera [60,61]. These four taxa are grouped in two pairs [58,59] (summarized in Table 1; for more detailed descriptions see S1 Appendix): (i) *D. glaciale* (Wulf.) Nyman subsp. *calcareum* (Vierh.) Hegi from the northeastern-most Alps and the parapatric *D. glaciale* (Wulf.) Nyman subsp. *glaciale* from the eastern Alps (Fig. 1, S1 Appendix) are diploid (2*n* = 60) and basiphilous growing on calcareous or, in case of *D. glaciale* subsp. *glaciale*, also on base-rich siliceous substrate; (ii) *D. clusii* (All.) Tausch s.s. (i.e., excluding *D. stiriacum* (Vill.) Dalla Torre) from the Alps (except the easternmost parts) and the allopatric *D. stiriacum* from the easternmost Central Alps and the Carpathians (Fig. 1, S1 Appendix) are acidophilic, but differ in being diploid (2*n* = 60) and tetraploid (2*n* = 120), respectively. Whereas a geographically restricted contact zone with morphologically intermediate individuals links *D. glaciale* subsp. *calcareum* and *D. glaciale* subsp. *glaciale* (Fig. 1; S1 Appendix), putative hybrids between *D. clusii* s.s. and *D. glaciale* subsp. *glaciale* occur throughout their overlapping distribution ranges in the eastern Central Alps (Fig. 1; S1 Appendix). The Alpine distribution ranges of both *D. glaciale* subsp. *calcareum* and *D. stiriacum* overlap with Pleistocene refugia [4,5,38] suggesting that their differentiation might be connected to this period.

**Table 1:** Overview of the taxa of the *Doronicum clusii* aggregate and their diagnostic morphological characters (summarized from [50,58,59,113,114,116]).

*D. glaciale* s.l.		*D. clusii* s.l.	
*D. glaciale* subsp. *calcareum* (= *D. calcareum*)	*D. glaciale* subsp. *glaciale* (= *D. glaciale* s.s.)	*D. clusii* s.s.	*D. stiriacum* (= *D. clusii* subsp. ‘*villosum*’)
margins of basal leaves scarcely hirsute (acute, stiff, clearly multiseriate eglandular trichomes 0.5–1.2 mm long)		margins of basal leaves pubescent (tangled, hyaline, uniseriate or indistinctly multiseriate eglandular trichomes >1 mm) (S1 Appendix), mostly eglandular, rarely with a few stipitate glands	
margins of basal leaves without glands (S1 Appendix); involucrum with glandular hairs 1–2 mm long, glands 0.3–0.5 mm long	margins of basal leaves with numerous short-stalked glands (S1 Appendix); involucrum with glandular hairs 0.5–1 mm long, glands sessile or very short	basal leaves tender, almost glabrous on the upper side; abundant glands on involucrum (S1 Appendix) and scape; corolla tubes of ray florets glabrous; corolla of ray florets 14–22 mm long	basal leaves thick, coarse, densely villous on both sides; sparse glands on involucrum (S1 Appendix) and scape; corolla tubes of the ray florets villous; corolla of ray florets 20–27 mm long

**Table 2:** Populations of the *Doronicum* taxa investigated, sampling locations (voucher information), relative fluorescence, number of investigated individuals, AFLP-derived gene diversity and rarity, Prabclus assignments, plastid haplotype, and GenBank accession numbers.

Pop. No.	Geographic origin^a^; voucher information	G1 peak ratio^b^	N_DAPI/AFLP_ ^c^	Genetic diversity	Rarity	Prabclus^d^	h_cp_ ^e^	Genbank accession numbers^f^
***D. glaciale* subsp. *calcareum* (= *D. calcareum*)**								
1	A: Rax-Schneeberg-Gruppe, Schneeberg; 15°48′27"E, 47°47′10"N, leg. CP & AH; WU: CP1000, http://herbarium.univie.ac.at/database/detail.php?ID=546203	1.135	5/5	0.093	1.82	calc: 5 (0.91–0.95; 0.00–0.01; 0.04–0.09)	h1	KP133673; KP133797; KP133735; KP133546
2	A: Mürzsteger Alpen, Schneealpe; 15°36′38"E, 47°41′49"N, leg. CP & GMS; WU: CP1001, http://herbarium.univie.ac.at/database/detail.php?ID=546199	1.140	5/4	0.119	1.45	calc: 4 (0.86–0.94; 0.00–0.01; 0.01–0.06)	h1	KP133674; KP133798; KP133736; KP133547
3	A: Mürzsteger Alpen, Gippel; 15°35′44"E, 47°47′59"N, leg. CP & JP; WU: CP1002, http://herbarium.univie.ac.at/database/detail.php?ID=546682	1.140	5/5	0.073	1.43	calc: 5 (0.86–0.94; 0.00–0.01; 0.05–0.10)	h2	KP133675; KP133799; KP133737; KP133548
4	A: Mürzsteger Alpen, Veitsch; 15°24′48"E, 47°39′52"N, leg. CP & HPG; WU: CP1003, http://herbarium.univie.ac.at/database/detail.php?ID=546683	1.157	5/4	0.093	1.77	calc: 4 (0.86–0.91; 0.00–0.01; 0.02–0.12)	h4	KP133676; KP133800; KP133738; KP133549
5	A: Ybbstaler Alpen, Ötscher; 15°12′07"E, 47°51′43"N, leg. R. Hehenberger; WU: CP1004, http://herbarium.univie.ac.at/database/detail.php?ID=546684	0.324*	5/5	0.109	1.80	calc: 3 (0.65–0.94; 0.00; 0.04–0.23); glac: 2 (0.53–0.56; 0.01; 0.14–0.17)	h2	KP133677; KP133801; KP133739; KP133550
**‘intermediate’: morphological intermediates between *D. glaciale* subsp. *calcareum* and *D. glaciale* subsp. *glaciale***								
6	A: Grazer Bergland, Hochlantsch; 15°25′25"E, 47°21′47"N, leg. CP; WU: CP1005, http://herbarium.univie.ac.at/database/detail.php?ID=546685	0.358*	5/5	0.102	1.22	calc: 4 (0.70–0.93; 0.00–0.01; 0.02–0.23); glac: 1 (0.32; 0.00; 0.13)	h1	KP133678; KP133802; KP133740; KP133551
7	A: Hochschwab-Gruppe, Mieserkogel; 15°15′34"E, 47°38′17"N, leg. CP & HPG; WU: CP1006, http://herbarium.univie.ac.at/database/detail.php?ID=546687	1.165	5/5	0.108	1.36	calc: 5 (0.56–0.93; 0.00–0.01; 0.04–0.25)	h1	KP133679; KP133803; KP133741; KP133552
8	A: Hochschwab-Gruppe, Obere Dullwitz; 15°09′37"E, 47°36′53"N, leg. CP & HPG; WU: CP1007, http://herbarium.univie.ac.at/database/detail.php?ID=546688	1.157	5/4	0.137	1.49	calc: 2 (0.78–0.80; 0.00; 0.09–0.13); glac: 2 (0.37–0.54; 0.00–0.01; 0.06–0.22a)	h1	KP133680; KP133804; KP133742; KP133553
9	A: Hochschwab-Gruppe, Hirschgrube; 15°05′20"E, 47°36′13"N, leg. CP & HPG; WU: CP1008, http://herbarium.univie.ac.at/database/detail.php?ID=546689	1.177	5/4	0.082	1.80	calc: 4 (0.42–0.81; 0.01; 0.09–0.58)	h5	KP133681; KP133805; KP133743; KP133554
10	A: Hochschwab-Gruppe, Kleiner Ebenstein; 15°01′29"E, 47°36′03"N, leg. CP & HPG; WU: CP1009, http://herbarium.univie.ac.at/database/detail.php?ID=546690	1.178	5/5	0.088	0.75	glac: 5 (0.53–0.73; 0.01; 0.00–0.03)	h3	KP133682; KP133806; KP133744; KP133555
***D. glaciale* subsp. *glaciale* (= *D. glaciale* s.s.)**								
11	A: Hochschwab-Gruppe, Polster; 14°57′39"E, 47°31′57"N, leg. CP & HPG; WU: CP1010, http://herbarium.univie.ac.at/database/detail.php?ID=546691	1.163	5/5	0.068	0.68	glac: 5 (0.49–0.68; 0.00–0.02; 0.03–0.11)	h1	KP133683; KP133807; KP133745; KP133556
12	A: Triebener Tauern, Gamskogel; 14°33′05"E, 47°22′02"N, leg. GMS; WU: 12257, http://herbarium.univie.ac.at/database/detail.php?ID=546692	1.172	5/5	0.078	0.66	glac: 5 (0.56–0.75; 0.00–0.02; 0.00–0.18)	h1	KP133684; KP133808; KP133746; KP133557
13	A: Wölzer Tauern, Schießeck; 14°19′20"E, 47°16′40"N, leg. PS & GMS; WU: 12295, http://herbarium.univie.ac.at/database/detail.php?ID=546693	1.156	5/5	0.073	0.59	glac: 5 (0.74–0.75; 0.00–0.01; 0.00–0.01)	h1	KP133685; KP133809; KP133747; KP133558
14	A: Karawanken, Hochstuhl; 14°10′20"E, 46°26′05"N, leg. BF & PS; WU: 12259, http://herbarium.univie.ac.at/database/detail.php?ID=546694	1.178	4/4	0.073	0.64	glac: 4 (0.74–0.75; 0.00–0.02; 0.00–0.02)	h2	KP133686; KP133810; KP133748; KP133559
15	A: Gurktaler Alpen, Gruft; 13°54′07"E, 46°54′19"N, leg. CP; WU: CP1011, http://herbarium.univie.ac.at/database/detail.php?ID=546695	1.169	5/5	0.063	0.84	glac: 5 (0.72–0.74; 0.00–0.01; 0.00–0.03)	h2	KP133687; KP133811; KP133749; KP133560
16	A: Schladminger Tauern, Höchstein; 13°47′29"E, 47°20′48"N, leg. GMS; WU: 12276, http://herbarium.univie.ac.at/database/detail.php?ID=546697	1.138	3/5	0.076	0.66	glac: 5 (0.70–0.75; 0.00–0.02; 0.00–0.03)	h2	KP133688; KP133812; KP133750; KP133561
17	I: Alpi Giulie, Ponza Grande; 13°41′51"E, 46°27′35"N, leg. BF & PS; WU: 12410, http://herbarium.univie.ac.at/database/detail.php?ID=546698	1.147	5/5	0.078	0.73	glac: 5 (0.67–0.75; 0.00–0.02; 0.00–0.06)	h2	KP133689; KP133813; KP133751; KP133562
18	A: Schladminger Tauern, Großes Gurpitscheck; 13°36′51"E, 47°12′35"N, leg. CP; WU: CP1012, http://herbarium.univie.ac.at/database/detail.php?ID=546699	1.198	5/5	0.013	0.56	glac: 5 (0.71–0.73; 0.03–0.04; 0.00)	h2	KP133690; KP133814; KP133752; KP133563
19	A: Dachsteinmassiv, Gosaukamm; 13°30′45"E, 47°30′50"N, leg. CP; WU: CP1013, http://herbarium.univie.ac.at/database/detail.php?ID=546700	1.156	5/5	0.057	0.66	glac: 5 (0.72–0.74, 0.01–0.02; 0.00–0.01)	h2	KP133691; KP133815; KP133753; KP133564
20	A: Radstädter Tauern, Draugstein; 13°17′17"E, 47°12′10"N, leg. CP & JP; WU: CP1014, http://herbarium.univie.ac.at/database/detail.php?ID=546701	1.161	5/5	0.058	0.66	glac: 5 (0.74–0.75; 0.01; 0.00)	h2	KP133692; KP133816; KP133754; KP133565
21	A: Kreuzeck-Gruppe, Salzkofel; 13°15′24"E, 46°50′54"N, leg. GMS; WU: 12235, http://herbarium.univie.ac.at/database/detail.php?ID=546702	1.178	5/5	0.062	0.54	glac: 5 (0.72–0.75; 0.00–0.02; 0.00–0.01)	h2	KP133693; KP133817; KP133755; KP133566
22	A: Goldberg-Gruppe, Trögereck; 12°54′53"E, 47°00′58"N, leg. CP; WU: CP1015, http://herbarium.univie.ac.at/database/detail.php?ID=546703	1.159	5/4	0.084	0.64	glac: 4 (0.73–0.75; 0.01–0.02; 0.00–0.01)	h2	KP133694; KP133818; KP133756; KP133567
23	I: Alpi Carniche, Monte Peralba; 12°43′30"E, 46°37′55"N, leg. RF & CG; WU: CP1016, http://herbarium.univie.ac.at/database/detail.php?ID=546704	1.148	5/5	0.082	0.55	glac: 5 (0.71–0.74; 0.00–0.02; 0.00–0.04)	h2	KP133695; KP133819; KP133757; KP133568
24	A: Glockner-Gruppe, Krefelder Hütte; 12°42′05"E, 47°12′46"N, leg. CP; WU: CP1017, http://herbarium.univie.ac.at/database/detail.php?ID=546705	1.170	5/5	0.033	0.57	glac: 5 (0.74–0.75; 0.01–0.02; 0.00)	h2	KP133696; KP133820; KP133758; KP133569
***D*. × *bauhini* (= *D. clusii* s.s. × *D. glaciale* subsp. *glaciale*)**								
25	A: Zillertaler Alpen, Plauener Hütte; 12°05′23"E, 47°07′06"N, leg. CP; WU: CP1018, http://herbarium.univie.ac.at/database/detail.php?ID=547698	1.111	5/5	0.065	0.73	–	h12	KP133697; KP133821; KP133759; KP143996 / KP133570–KP133579
26	A: Tuxer Alpen, Klammspitzen; 11°36′59"E, 47°09′52"N, leg. CP & JP; WU: CP1019, http://herbarium.univie.ac.at/database/detail.php?ID=546708	1.127	5/5	0.061	0.48	–	h2	KP133698; KP133822; KP133760; KP143997 / KP133580–KP133583
***D. stiriacum***								
27	A: Lavanttaler Alpen, Zirbitzkogel; 14°33′26"E, 47°04′05"N, leg. CP; WU: CP1020, http://herbarium.univie.ac.at/database/detail.php?ID=546710	2.093	5/5	0.099	1.58	stir: 5 (0.74–0.90; 0.00–0.01; 0.10–0.26)	h17	KP133699; KP133823; KP133761; KP133584
28	A: Triebener Tauern, Geierkogel; 14°29′59"E, 47°24′00"N, leg. GMS; WU: 12243, http://herbarium.univie.ac.at/database/detail.php?ID=546712	–	0/4	0.127	2.05	stir: 4 (0.87–0.91; 0.00–0.02; 0.10–0.12)	h18	KP133700; KP133824; KP133762; KP133585
29	A: Rottenmanner Tauern, Großer Bösenstein; 14°24′46"E, 47°26′22"N, leg. PEG; WU: CP1021, http://herbarium.univie.ac.at/database/detail.php?ID=546714	2.029	5/5	0.134	1.79	stir: 5 (0.64–0.91; 0.00–0.01; 0.09–0.36)	h18	KP133701; KP133825; KP133763; KP133586
30	A: Wölzer Tauern, Hoher Zinken; 14°20′30"E, 47°16′14"N, leg. CP; WU: CP1022, http://herbarium.univie.ac.at/database/detail.php?ID=546715	2.004	5/5	0.113	1.81	stir: 5 (0.88–0.91; 0.00–0.01; 0.09–0.12)	h20	KP133702; KP133826; KP133764; KP143998 / KP133587–KP133589
31	A: Wölzer Tauern, Jochspitze; 14°11′36"E, 47°23′31"N, leg. CP; WU: CP1023, http://herbarium.univie.ac.at/database/detail.php?ID=546717	1.998	5/5	0.118	1.81	stir: 5 (0.87–0.90; 0.00–0.01; 0.10–0.13)	h19	KP133703; KP133827; KP133765; KP143999 / KP133590–KP133602
32	A: Gurktaler Alpen, Kaserhöhe; 13°55′05"E, 46°54′51"N, leg. PS; WU: CP1024, http://herbarium.univie.ac.at/database/detail.php?ID=546718	2.059	3/4	0.013	2.41	stir: 4 (0.66–0.69; 0.16–0.17; 0.15–0.17)	h18	KP133704; KP133828; KP133766; KP144000 / KP133603–KP133614
33	A: Schladminger Tauern, Preber; 13°51′52"E, 47°13′10"N, leg. PEG; WU: CP1025, http://herbarium.univie.ac.at/database/detail.php?ID=546724	2.190	5/5	0.110	2.15	stir: 5 (0.86–0.90; 0.00–0.01; 0.100.15)	h20	KP133705; KP133829; KP133767; KP133615
34	A: Schladminger Tauern, Höchstein; 13°47′13"E, 47°20′45"N, leg. GMS; WU: 12275, http://herbarium.univie.ac.at/database/detail.php?ID=548652	2.047	5/5	0.108	1.34	stir: 5 (0.85–0.91; 0.00–0.01; 0.10–0.15)	h18	KP133706; KP133830; KP133768; KP133616
35	A: Schladminger Tauern, Großes Gurpitscheck; 13°36′50"E, 47°12′35"N, leg. CP; WU: CP1026, http://herbarium.univie.ac.at/database/detail.php?ID=548654	1.966	5/5	0.026	1.41	stir: 5 (0.67–0.78; 0.06–0.11; 0.13–0.28)	h18	KP133707; KP133831; KP133769; KP144001 / KP133616–KP133621
36	RO: Munţii Rodnei, Pietros; 24°38′17"E, 47°35′43"N, leg. M. Puşcaş; WU: s.n., http://herbarium.univie.ac.at/database/detail.php?ID=548655	2.108	5/5	0.087	1.90	stir: 5 (0.56–0.74; 0.09–0.12; 0.16–0.35)	h15	KP133708; KP133832; KP133770; KP144002 / KP133621–KP133627
37	SK: Vysoké Tatry, Čierny Štit; 20°12′09"E, 49°12′19"N, leg. A. & M. Ronikier; WU: CP200, http://herbarium.univie.ac.at/database/detail.php?ID=548656	2.124	6/6	0.090	2.25	stir: 6 (0.71–0.75; 0.11–0.13; 0.14–0.18)	h16	KP133709; KP133833; KP133771; KP1440003 / KP133628–KP133638
38	SK: Nízke Tatry, Ďumbier; 19°38′06"E, 48°56′25"N, leg. V. Kolarčik; WU: CP199, http://herbarium.univie.ac.at/database/detail.php?ID=550244	2.002	5/4	0.095	3.21	stir: 4 (0.67–0.75; 0.09–0.12; 0.16–0.23)	h14	KP133710; KP133834; KP133772; KP144004 / KP133638–KP133648
***D. clusii* s.s.**								
39	A: Kreuzeckgruppe, Zietenkopf; 12°56′18"E, 46°48′33"N, leg. CP; WU: CP1027, http://herbarium.univie.ac.at/database/detail.php?ID=550245	1.153	5/5	0.024	0.56	clus: 5 (0.82–0.97; 0.03–0.16; 0.00–0.03)	h13	KP133711; KP133835; KP133773; KP133649
40	A: Villgratner Berge, Thurntaler; 12°23′06"E, 46°46′36"N, leg. CP; WU: CP1028, http://herbarium.univie.ac.at/database/detail.php?ID=550246	0.351*	5/5	0.035	0.72	clus: 5 (0.96–0.99; 0.02–0.05; 0.00)	h11	KP133712; KP133836; KP133774; KP133650
41	A: Zillertaler Alpen, Sonntaglahnerkopf; 12°05′32"E, 47°07′04"N, leg. CP; WU: CP1029, http://herbarium.univie.ac.at/database/detail.php?ID=550247	1.181	4/3	0.006	1.28	clus: 3 (0.97; 0.04; 0.00)	h9	KP133713; KP133837; KP133775; KP133651
42	I: Dolomiten, Plose; 11°43′56"E, 46°42′19"N, leg. CP & AH; WU: CP1030, http://herbarium.univie.ac.at/database/detail.php?ID=550248	1.147	5/5	0.049	0.78	clus: 5 (0.94–0.99; 0.02–0.07; 0.00)	h12	KP133714; KP133838; KP133776; KP133652
43	I: Dolomiti, Cima d’Asta; 11°36′28″E, 46°10′35″N, leg. RF; WU: CP1031, http://herbarium.univie.ac.at/database/detail.php?ID=550249	1.173	5/5	0.061	0.77	clus: 5 (0.96–0.99; 0.02–0.04; 0.00)	h8	KP133715; KP133839; KP133777; KP133653
44	A: Tuxer Alpen, Patscherkofel; 11°28′30"E, 47°12′45"N, leg. BF & PS; WU: 12272, http://herbarium.univie.ac.at/database/detail.php?ID=550841	0.340*	5/5	0.056	0.73	clus: 5 (0.95–1.00; 0.00–0.05; 0.00–0.01)	h12	KP133716; KP133840; KP133778; KP133654
45	A: Stubaier Alpen, Rietzer Grieskogel; 11°03′20"E, 47°14′50"N, leg. BF & PS; WU: 12266, http://herbarium.univie.ac.at/database/detail.php?ID=550842	1.149	5/5	0.047	0.79	clus: 5 (0.98–0.99; 0.01–0.02; 0.00–0.01)	h12	KP133717; KP133841; KP133779; KP133655
46	A: Ötztaler Alpen, Hohe Aifner Spitze; 10°43′45"E, 47°06′10"N, leg. BF & PS; WU: 12264, http://herbarium.univie.ac.at/database/detail.php?ID=550843	1.135	5/5	0.063	0.68	clus: 5 (0.93–0.98; 0.02–0.07; 0.00–0.01)	h10	KP133718; KP133842; KP133780; KP133656
47	I: Gruppo dell’ Ortles—Cevedale, Passo dei Contrabbandieri; 10°34′23″E, 46°17′17"N, leg. MS; WU: CP1032, http://herbarium.univie.ac.at/database/detail.php?ID=550844	1.180	5/5	0.056	0.73	clus: 5 (0.91–1.00; 0.01–0.08; 0.00–0.02)	h8	KP133719; KP133843; KP133781; KP133657
48	A: Silvretta, Hohes Rad; 10°06′35"E, 46°54′35"N, leg. BF & PS; WU: 12292, http://herbarium.univie.ac.at/database/detail.php?ID=550845	1.179	5/5	0.058	0.84	clus: 5 (0.94–1.00; 0.01–0.06; 0.00)	h8	KP133720; KP133844; KP133782; KP133658
49	CH: Albula-Alpen, Flüela Schwarzhorn; 09°56′41"E, 46°43′49"N, leg. MS; WU: CP1033, http://herbarium.univie.ac.at/database/detail.php?ID=550846	1.149	6/6	0.056	1.09	clus: 6 (0.94–0.98; 0.02–0.07; 0.00)	h6	KP133721; KP133845; KP133783; KP133659
50	I: Alpi del Bernina, Bocchetta delle Forbici; 09°54′01"E, 46°19′55"N, leg. MS; WU: CP1034, http://herbarium.univie.ac.at/database/detail.php?ID=550847	1.122	5/5	0.063	0.93	clus: 5 (0.93–0.99; 0.02–0.06; 0.00–0.02)	h10	KP133722; KP133846; KP133784; KP133660
51	CH: Glarner Alpen, Pizol; 09°17′53"E, 46°56′29"N, leg. BF & PS; WU: 12288, http://herbarium.univie.ac.at/database/detail.php?ID=550848	1.163	4/4	0.047	0.90	clus: 4 (0.97–0.99; 0.02–0.04; 0.00)	h10	KP133723; KP133847; KP133785; KP133661
52	CH: Gotthardmassiv, Stotzigen Firsten; 08°25′55"E, 46°33′52"N, leg. CP & MG; WU: CP1035, http://herbarium.univie.ac.at/database/detail.php?ID=550849	1.139	5/5	0.061	1.10	clus: 5 (0.98–0.99; 0.01–0.03; 0.00)	h7	KP133724; KP133848; KP133786; KP133662
53	CH: Alpes valaisannes, Montagne d’Arolla; 07°26′42"E, 46°00′58"N, leg. CP & MG; WU: CP1036, http://herbarium.univie.ac.at/database/detail.php?ID=550850	1.123	5/5	0.050	1.15	clus: 5 (0.98–0.99; 0.02; 0.00)	h7	KP133725; KP133849; KP133787; KP133663
***D. grandiflorum***								
54	A: Ybbstaler Alpen, Dürrenstein; 15°03′22"E, 47°47′11"N, leg. CP & HPG; WU: CP228, http://herbarium.univie.ac.at/database/detail.php?ID=387276	1.050	5/3	0.036	1.03	–	h23	KP133726; KP133850; KP133788; KP133664
55	A: Dachsteinmassiv, Krippenstein; 13°41′33"E, 47°31′14"N, leg. CP; WU: CP223, http://herbarium.univie.ac.at/database/detail.php?ID=387285	0.331*	5/3	0.054	0.93	–	h21	KP133727; KP133851; KP133789; KP133665
56	I: Alpi Carniche, Monte Peralba; 12°43′41"E, 46°37′26"N, leg. RF & CG; WU: CP229, http://herbarium.univie.ac.at/database/detail.php?ID=387309	1.055	5/5	0.082	0.55	–	h23	KP133728; KP133852; KP133790; KP133666
57	I: Alpi Orobie Orientali, Lago di Coca; 10°00′04"E, 46°03′40"N, leg. MS; WU: CP224, http://herbarium.univie.ac.at/database/detail.php?ID=387310	0.324*	5/5	0.031	0.86	–	h21	KP133729; KP133853; KP133791; KP133667
58	I: Alpi Cozie, Bivacco Bonelli; 06°55′54"E, 44°28′03"N, leg. A. Tribsch; SZU: 111714, http://herbarium.univie.ac.at/database/detail.php?ID=546681	1.088	5/5	0.041	0.87	–	h22	KP133730; KP133854; KP133792; KP133668
59	BIH: Čvrsnica, Pločno; 17°34′23"E, 43°36′04"N, leg. RF & D. Reich; WU: CP196, http://herbarium.univie.ac.at/database/detail.php?ID=311979	–	–	–	–	–	h23	KP133731; KP133855; KP133793; KP133669
60	BIH: Prenj, Zelena glava; 17°54′05"E, 43°33′01"N, leg. PS, BF & D. Kutnjak; WU: 12861, http://herbarium.univie.ac.at/database/detail.php?ID=311978	–	–	–	–	–	h23	KP133732; KP133856; KP133794; KP133670
***D. austriacum***								
61	A: Fischbacher Alpen, Stuhleck; 15°48′11"E, 47°34′19"N, leg. CP & GMS; WU: CP256, http://herbarium.univie.ac.at/database/detail.php?ID=387671	–	–	–	–	–	h25	KP133733; KP133857; KP133795; KP133671
***D. carpaticum* (= *D. columnae* subsp. *carpaticum*)**								
62	RO: Munţii Făgăraş, Cabana Paltinu; 24°36′52"E, 45°36′19"N, leg. CP; WU: CP204, http://herbarium.univie.ac.at/database/detail.php?ID=387667	–	–	–	–	–	h24	KP133734; KP133858; KP133796; KP133672

^a^ countries: A, Austria; BIH, Bosnia and Herzegovina; CH, Switzerland; I, Italy; RO, Romania; SK, Slovakia; collectors: PEG, P. Escobar García; AH, RF, R. Flatscher; BF, B. Frajman; CG, C. Gilli, MG, M. Gina; HPG, H. P. Grohmann; AH, A. Hahnekamp; CP, C. Pachschwöll; JP, J. Pachschwöll; GMS, G. M. Schneeweiss, PS, P. Schönswetter; MS, M. Sonnleitner.

^b^ Estimated with flow cytometry for a pooled sample of 3–6 individuals and given as ratio of DAPI-stained nuclei compared to the internal standards *Pisum sativum* (not indicated) or *Vicia faba* (marked with an asterisk).

^c^ Number of individuals used for DAPI flow cytometry and AFLP genotyping, respectively.

^d^ Plastid DNA haplotypes derived from *ndh*F-*rpl*32, *rpl*32-*trn*L_UAG_ and 3′*rps*16–5′*trn*K_UUU_-sequences.

^e^ Assignment to the four Prabclus groups. calc: *D. glaciale* subsp. *calcareum*, glac: *D. glaciale* subsp. *glaciale*, clus: *D. clusii* s.s., stir: *D. stiriacum*: number of individuals (proportion of correct assignment to the original cluster, i.e. bootstrap support of correct assignment; proportion of assignment to additional clusters within the same species, i.e., oversplitting; proportion of no assignment).

^f^ *ndh*F-*rpl*32; *rpl*32-*trn*L_UAG_; 3′*rps*16–5′*trn*K_UUU_; ITS uncloned / ITS cloned.

Here, we investigate diversification and its underlying causes in the *D. clusii* aggregate. To this end, we use Amplified Fragment Length Polymorphisms (AFLPs), nuclear and plastid DNA sequences, DNA ploidy level estimation by flow cytometry and chromosome counts. AFLPs are rapidly homogenizing, biparentally inherited markers well suited to resolve relationships among closely related taxa because of their ability to provide phylogenetic signal in young or rapidly evolving study systems (e.g., [62,63]). Biparentally inherited nuclear Internal Transcribed Spacer (ITS) sequences are, despite a number of potential problems [64], appropriate for addressing evolutionary questions at the species level including hybridization [65] and have been widely used in Asteraceae including *Doronicum* [49,52]. Maternally inherited (for *Doronicum* and other Asteraceae [66]) plastid DNA sequences have slower mutation rates [67,68] and were widely and successfully applied for phylogenetic and phylogeographic analyses and, together with nuclear ITS data, inference of reticulate relationships also in *Doronicum* [49,52]. Flow cytometry can be used to rapidly screen ploidy levels (calibrated by chromosome counts) for numerous samples [69,70].

The main goal of the present study is to determine phylogenetic relationships among the four members of the *D. clusii* aggregate (only *D. clusii* s.s. and *D. glaciale* subsp. *glaciale* have been included by [49]) and to unravel evolutionary patterns within the aggregate. We aim to answer the following questions. (1) Do the traditionally recognized taxa *D. clusii* s.s., *D. glaciale* subsp. *calcareum*, *D. glaciale* subsp. *glaciale*, and *D. stiriacum* constitute genetically distinct lineages? Are morphologically intermediate forms between *D. glaciale* subsp. *calcareum* and *D. glaciale* subsp. *glaciale* and between *D. clusii* s.s. and *D. glaciale* subsp. *glaciale* (i.e., *D. × bauhini* Saut.) of hybrid origin? (2) Are populations of *D. stiriacum* in the eastern Alps tetraploid, as are those in the Carpathians? Is tetraploid *D. stiriacum* of auto- or allotetraploid origin? (3) Do patterns of genetic diversity and/or rarity reflect putative Pleistocene refugia previously suggested for acidophilic and basiphilous species [5]?

## Materials and Methods

### Ethics Statement

The investigated taxa are neither endangered nor protected except at the following locations: *D. stiriacum* pop. 32 (collected under permit 8-NAT-259/3/2004 from the Amt der Kärntner Landesregierung, Austria); *D. stiriacum* pop. 36 (collected under permit from the Parcul Național Munții Rodnei, Romania); *D. stiriacum* pop. 37 (collected under permit no. 1762/565/04–5.1 from the Ministerstvo Životneho Prostredia, Slovakia); *D. stiriacum* pop. 38 (collected under permit no. 6878/2008–2.1 from the Ministerstvo Životneho Prostredia, Slovakia).

### Plant Material

Sampling focused on the eastern Alps where all four taxa of the *D. clusii* aggregate occur (Fig. 1). Our sampling of *D. glaciale* subsp. *calcareum*, *D. glaciale* subsp. *glaciale* and *D. stiriacum* is comprehensive and covers the species’ entire distribution areas. As our study did not primarily focus on intraspecific phylogeography—a wealth of data already exists for the Alps (reviewed in [5,71])—we sampled *D. clusii* s.s. westwards only to southern Switzerland. As outgroups, the closely related *D. grandiflorum* Lam. (Alps and southern European mountain ranges [50,72]) and, for DNA sequences only, the more distantly related [49] *D. austriacum* Jacq. and *D. carpaticum* (Griseb. & Schenk) Nyman (= *D. columnae* Ten. subsp. *carpaticum* (Griseb. & Schenk) Soó [54]) were included. Leaf material from three to six (median five) individuals per population was collected in the field mostly between 2008 and 2010 (Table 2) and immediately dried in silica gel. Herbarium vouchers of each population were databased and deposited at the herbarium of the University of Vienna (WU; http://herbarium.univie.ac.at/) and at the herbarium of the University of Salzburg (SZU).

### DNA extraction

Total genomic DNA was extracted from c. 10 mg tissue following [73,74] with the modifications detailed in [18]. The quality of the extracted DNA was checked on 1% TAE-agarose gels and quantified with a Nanodrop Spectrophotometer ND-100 (PEQLAB, Erlangen, Germany).

### Flow Cytometry and Chromosome Counts

DNA ploidy levels [75] of silica gel-dried leaf tissue were determined using 4′,6-diamidino-2-phenylindole (DAPI) flow cytometry as described in [76]. Measurements of DAPI-stained nuclei were performed for the same individuals used for AFLPs plus four additional specimens (total of 280 individuals; Table 2). In a first round, one individual per population was measured to assess putative DNA ploidy variation within a given taxon. In a second round, which was preceded by initial tests proving that a single individual with deviating ploidy could be detected if co-analysed with five individuals of another ploidy, all individuals of a population were pooled to test the possible presence of minority ploidy levels; this approach does not affect the reliability of the ploidy estimates [44,76–78]. Following [76], *Pisum sativum* ‘Ctirad’ (1C = 4.55 pg [79]) and *Vicia faba* ‘Inovec’ (1C = 13.45 pg [80]) were used as internal standards, and fluorescence intensities of 3000 particles were recorded.

Actively growing root meristems were obtained from individuals that were collected in the field (along with the silica samples, but not used in the genetic analyses) and subsequently cultivated in the Botanical Garden of the University of Vienna (HBV), or from seedlings (pop. 35 only). After initial experiments, these meristems were pre-treated with 0.002 M 8-hydroxyquinoline for 2 h at room temperature and for 2 h at 4°C (putative diploids) and for 3.5 at room temperature and for 4.5 h at 4°C (putative tetraploids), respectively, in darkness; subsequently they were fixed in 3: 1 ethanol: acetic acid and stored at-20°C until use. Fixed meristems were hydrolysed in 5N HCl for 30 min at room temperature, rinsed with tap water, and stained with Schiff’s reagent (Merck, Vienna, Austria) in darkness for 60 min. Squash preparations were made in a drop of 60% acetic acid. Preparations were analysed with an Axioplan2 microscope (Carl Zeiss, Vienna, Austria). Images were acquired with a CCD camera and processed using Axiovision 4.8 (Carl Zeiss). Image quality was optimised using Adobe Photoshop CS3 (Adobe Systems, San Jose, CA, USA) with options that applied uniformly to all pixels of the image.

### AFLPs

AFLP data were generated for taxa of the *D. clusii* aggregate and for *D. grandiflorum* populations from the Alps. The AFLP procedure followed [81] with the modifications described in [82] using approximately 150–500 ng of total DNA. For the selective amplification initially 24 selective primer combinations with three or four *Mse*I selective bases were screened. The three final primer combinations were (fluorescent dye in parentheses): *Eco*RI (6-Fam)-ACA/*Mse*I-CACC, *Eco*RI (VIC)-ACG/*Mse*I-CAAG, and *Eco*RI (NED)-ACA/*Mse*I-CAAG. In each PCR plate, the same seven individuals were replicated to calculate the error rate according to [83], and to exclude non-reproducible fragments from the analysis. Thirty-two between-plate replicates were successfully amplified, and ten individuals were extracted twice. From the restriction-ligation onwards, one blank sample per plate was included to test for systematic contamination.

### DNA sequences

One individual per population (usually one that was also included in the AFLP data set) was used for generating DNA sequences. In addition, for DNA sequences up to five individuals per population were sequenced in 28 randomly selected populations to check for possible intra-population variation.

For plastid markers, the following regions were screened using eight individuals from different taxa: *pet*L-*psb*E, *psb*J-*pet*A, 3′*trn*V_UAC_-*ndh*C, *psb*D-*trn*T_GGU_, *atp*I-*atp*H, *trn*Q_UUG_-5′*rps*16, 3′*rps*16–5′*trn*K_UUU_, the *ndh*A-intron, *ndh*F-*rpl*32, *rpl*32-*trn*L_UAG_ (all [67]), the *rps*16-intron [84], and the *trn*T_UGU_-*trn*F_GAA_ region [85]. Of these twelve markers, the three most variable ones (*ndh*F-*rpl*32, *rpl*32-*trn*L_UAG_, 3′*rps*16–5′*trn*K_UUU_) were selected. The PCR reaction mixes (totalling 15 μL) contained 4.8 μL REDTaq Ready Mix PCR reaction mix (Sigma-Aldrich, Vienna, Austria), 7.4 μL water, 0.6 μL BSA (10 mg/mL; Promega, Vienna, Austria), 0.3 μL of each primer (10 μM; Sigma-Aldrich) and 1 μL of genomic DNA. The *ndhF-rpl32* intergenic spacer region was amplified with the primers of [67], using the following PCR conditions: 1 min at 95°C; 10 cycles of 30 s at 95°C, 30 s at 47°C, 90 s at 65°C; 25 cycles of 30 s at 95°C, 30 s at 49°C, 90 s at 65°C; 8 min at 65°C. The 3′*rps*16–5′*trn*K_UUU_ intergenic spacer was amplified with newly designed primers (rps16-F-PD2: 5′-GTGGGTTTTTATGATCCGATCAAG-3′, trnK-R-PD2: 5′-TTAAAAGCCGAGTACTCTACCGTTG-3′), using the following PCR conditions: 1 min at 95°C; 35 cycles: 30 s at 95°C, 30 s at 56 or 63°C, 90 s at 72°; 8 min at 72°C. The *rpl*32-*trn*L_UAG_ intergenic spacer was amplified with newly designed primers (rpl32-F-PD2: 5′-AGGAAAGGATATTGGGCGGCG-3′, trnL-R-PD2: 5′-TTTCACCATAGCGGCTTGCTCG-3′), using the following PCR conditions: 1 min at 95°C, 35 cycles: 30 s at 95°C, 30 s at 63°C, 90 s at 72°; 8 min at 72°C. Samples for which the amplification of *ndh*F-*rpl*32 and *rpl*32-*trn*L_UAG_ repeatedly failed were amplified with AmpliTaq Gold in 12.5 μL reactions containing 0.1 μL AmpliTaq Gold DNA polymerase (Applied Biosystems, Foster City, CA), 0.1 μL BSA (1mg/mL; Promega), 7.8 μL water, 1.25 μL 10×AmpliTaq Gold buffer (Applied Biosystems), 1 μL dNTPs (10 mM; Applied Biosystems), 1.25 μL MgCl_2_ and 0.25 μL of the primer pairs, using the same PCR conditions.

The nuclear ITS region was amplified using the primers ITS101 (17SE) and ITS102 (26SE) of [86]. PCR reactions were performed in volumes of 15 μL comprising 0.5 μL of total genomic DNA, 4.8 μL REDTaq Ready Mix PCR reaction mix (Sigma-Aldrich), 7.4 μL water, 0.6 μL BSA (1 mg/mL; New England BioLabs, Ipswich, MA, USA) and 0.3 μL (10 μM) of the primer pairs. The PCR conditions were: 1 min at 95°C, 35 cycles with 30 s at 95°C, 30 s at 52°C, 90 s at 72°C followed by 7 min at 72°C. Critical samples that repeatedly failed were amplified in 12.5 μL reactions containing 0.1 μL AmpliTaq Gold DNA polymerase (Applied Biosystems), 0.1 μL BSA (1mg/mL; Promega), 7.8 μL water, 1.25 μL 10×AmpliTaq Gold buffer (Applied Biosystems), 1 μL dNTPs (10 mM; Applied Biosystems), 1.25 μL MgCl_2_ and 0.25 μL of the primer pairs. The PCR conditions were 10 s at 95°C, 35 cycles with 30 s at 94°C, 1 min at 52°C, 1 min at 72°C followed by 10 s at 72°. ITS sequences of the putative hybrid *D. × bauhini* (pops. 25, 26) and of a subset of *D. stiriacum* samples (pops. 30–32, 35–38), showing a significant amount of ambiguities in direct sequencing (Table 3), were cloned. Molecular cloning was done as described in [8] with the following modifications: inserts of 3–13 clones were amplified using the universal primers M13F(–47) and M13R(–48) and 1 μL of colony DNA re-suspended in 60 μL of double distilled water. For all PCR programs a ramp temperature of 1.2°C/s was used.

**Table 3:** Polymorphic sites from the ITS region in the *Doronicum clusii* aggregate: nucleotides are those found without cloning and (in parentheses) after cloning.

Taxon	Alignment position											
	072	116	120	132	152	156	305	488	551	629	661	688
*D. glaciale* subsp. *calcareum*	G	T	A	T	C	C	C	A	A	T	A	A
‘intermediate’ ^a^	G	T	A/R	T/Y	C	C	C	A	A	T	A	A
*D. glaciale* subsp. *glaciale*	G	T	A	T	C	C	C	A	A	T	A	A
*D. clusii* s.s.	T	C	G	C	T/Y	T	T	G	A	C	A	A
*D. stiriacum* (Alps)	G	C/T/Y (C/T)	G	C/Y (C/T)	C	C/T/Y (C/T)	C	A/G/R (A/G)	A/C/M (A/C)	C/Y (C/T)	A/G	A/G/R (A/G/R)
*D. stiriacum* (Carpathians)	G	C/Y (C/T)	G	C	C	T/Y (C/T)	C	G/R (A/G)	A	C	G/R (A/G)	A/R (A/G)
*D. × bauhini* (pop. 25)	T (G/T)	Y (C/T)	R (A/G)	Y (C/T)	C (C/T)	Y (C/T)	Y (C/T)	R (A/G)	A	Y (C/T)	A	A
*D. × bauhini* (pop. 26)	K (G/T)	Y (C/T)	R (A/G)	Y (C)	C	Y (C/T)	Y (C/T)	R (A/G)	A	Y (C/T)	A	A

^a^ morphological intermediates between *D. glaciale* subsp. *calcareum* and *D. glaciale* subsp. *glaciale*.

PCR products were purified using Exonuclease I and FastAP thermosensitive alkaline phosphatase (Fisher Scientific, St. Leon-Rot, Germany) following the manufacturer’s instructions. Cycle sequencing reactions were performed using 5 μL of purified template, 1 μL of primer (3.2 μM) and 1 μL BigDye Terminator (Applied Biosystems), cleaned with Sephadex G-50 Fine (GE Healthcare Bio-Sciences, Uppsala, Sweden) and sequenced on an ABI 3730 DNA Analyzer capillary sequencer (Applied Biosystems).

### AFLP Data Analyses

Raw AFLP data were aligned with the internal size standard using ABI Prism GeneScan 3.7.1 (Applied Biosystems), and imported into Genographer 1.6.0 [87] for scoring. Each AFLP fragment was scored using the ‘thumbnail’ option, which allows the comparison of the signal of each fragment (present or absent) over all samples. Unambiguous bands in the size range of 100–500 bp were scored and exported as a presence/absence matrix. Shorter fragments were not scored due to the more frequent occurrence of non-homologous fragments [88]. Nei’s gene diversity over loci [89] termed ‘genetic diversity’ in the following, and the frequency of rare markers as frequency-down-weighted marker values ([19,90]; ‘rarity 1’ in AFLPdat), termed ‘rarity’ in the following, were calculated for each population with the R script AFLPdat ver. 20.10.2010 [91]. Briefly, each marker is down-weighted by its frequency in the entire data set. Subsequently, the rarity for an individual is calculated as the sum of the (down-weighted) markers present in this individual; population rarity values are estimated as the average of the individuals’ rarity values.

Genetic diversity and rarity were compared (1) among the four constituents of the *D. clusii* aggregate, (2) between Alpine populations of *D. stiriacum* from glacial refugia and populations from glaciated areas and (3) between *D. glaciale* subsp. *calcareum* and *D. glaciale* subsp. *glaciale* excluding the morphologically intermediate populations 6–10. Statistical comparisons were done with SPSS 21 (SPSS, Chicago, IL, USA) using two-tailed t-tests for normally distributed data and Mann-Whitney U-tests for data without normal distribution. Normal distribution was tested for with the Kolmogorov-Smirnov test. Post-hoc Tukey-Kramer tests were applied to detect differences among and within taxonomic groups at a 5% confidence level, a frequently applied confidence level in similar comparisons (e.g., [92,93]).

A NeighborNet [94] was produced with SplitsTree 4.13.1 [95] using uncorrected P-distances. NeighborNets display congruent and conflicting signals in a data set as a series of splits, where the weight of a split is visible as the length of a line or a set of parallel lines on the diagram. They are well suited to display conflicting signals, as is expected to result from hybridization or introgression. Bootstrap support values were obtained from neighbour-joining trees using Nei-Li distances and 2,000 pseudoreplicates in SplitsTree.

For a data set comprising samples of the *D. clusii* aggregate only (excluding *D. grandiflorum*) excluding the hybrid *D. × bauhini* (see Results), groups were delimited using Gaussian clustering with a noise component for outliers as implemented in the R library Prabclus 2.2–4 [96]. This approach is recommended for dominant multilocus genetic data and results are considered comparable or superior to model-based approaches such as STRUCTURE [97]. Briefly, nonmetric multidimensional scaling (NMDS [98]) is performed on a distance-matrix. The resulting Euclidean variables are used to determine clusters of individuals based on Gaussian clustering where the number of clusters is determined by the Bayesian Information Criterion using the R functions prabinit (default settings, distance = Jaccard) and prabclus (settings: mdsmethod = ‘kruskal’, nclus = 0:6). As the default number of nearest neighbours to determine the initial noise estimation (nnk) of 7 (i.e., the number of individuals divided by 40 and rounded to the next-largest integer: [97: p. 492]) yielded meaningless results (random non-assignments over the whole data set), this parameter was finally set to 3, that is the number of individuals from the largest of the three distinct groups (i.e., the one containing *D. glaciale* subsp. *calcareum*, *D. glaciale* subsp. *glaciale* and their morphological intermediates) divided by 40 and rounded to the next-largest integer. The number of NMDS dimensions (mdsdim) was 3, which is the smallest mdsdim with a stress value below the arbitrary cut-value of 10% as determined by the function stressvals. Prabclus was repeated 200 times with a bootstrapped matrix (generated using seqboot from the PHYLIP package [99]) to test the support of group assignments. Bootstrap values of individuals were calculated as percentage of correct assignment to the original cluster (vs. non-assigned or assigned to another cluster). Two-dimensional visualization of the initial, non-bootstrapped matrix was performed with SigmaPlot 12.5 (Systat Software, San Jose, CA, USA).

For a data set containing *D. glaciale* subsp. *calcareum*, *D. glaciale* subsp. *glaciale* and their morphological intermediates (data set 1) and for a second data set containing populations of *D. clusii* s.s., *D. glaciale* subsp. *glaciale* and their putative hybrid *D. × bauhini* (data set 2), population structure was inferred employing a Bayesian clustering approach based on MCMC estimations developed for dominant markers with recessive alleles (STRUCTURE 2.3.3 [100,101]). Since we assumed admixture between *D. glaciale* subsp. *calcareum* and *D. glaciale* subsp. *glaciale* and between *D. clusii* s.s., *D. glaciale* subsp. *glaciale* and their putative hybrid, only the admixture model with correlated (data set 1, which comprises closely related populations; [102]) or uncorrelated allele frequencies (data set 2) was used. Ten replicate runs for each K (number of groups) ranging from 1 to 10 were carried out at the Bioportal of the University of Oslo (http://www.bioportal.uio.no), using a burn-in of 10^5^ iterations followed by 10^6^ additional MCMC iterations. Similarity among results of different runs for the same K was calculated according to [103,104] using the R-script Structure-sum ver. 2009 (part of AFLPdat [91]). The optimal number of groups was identified as the value of K where the likelihood started to flatten out, the results of replicate runs were identical, no empty groups were encountered and the mean delta K was at the maximum. DISTRUCT 1.1 [105] was used to display the results graphically.

### DNA Sequence Data Analyses

DNA sequences were assembled using SeqMan 7.0.0 (DNAStar, Madison, WI, USA) and manually edited and aligned with BioEdit 7.2.0 [106]. The alignment of the three concatenated plastid markers was analysed using statistical parsimony as implemented in TCS 1.21 [107] with the connection limit set to 95%; gaps were treated as fifth character state. For this analysis, indels longer than 1 bp and inversions were reduced to single base pair columns allowing those structural mutations to be counted as single base pair mutations only; additionally, mononucleotide repeats were removed due to their high degree of homoplasy at larger geographical scales [108]. As haplotype networks are known to be misled by ambiguous and missing data [109], ITS sequences (cloned ones in case of *D. × bauhini* and of *D. stiriacum* pops. 30–32 and 35–38) were analysed instead using maximum likelihood as implemented in RAxML 8.0.17 [110] using the GTRCAT substitution model and the fast bootstrap approach [111] with 500 replicates.

## Results

### Flow Cytometry and Chromosome Counts

CVs of G0/G1 peaks of the internal references standards were always below 3%. As expected, dehydrated samples showed higher CVs; the values usually did not exceed 5%, a threshold recommended by [69], but approached 7% in a few samples of the tetraploid *D. stiriacum*. It is known that polyploids often perform worse with DAPI in terms of quality and storability than diploids because nuclei may detoriorate faster [112]. DNA ploidy level measurements (Table 2) revealed that all individuals of *D. clusii* s.s., *D. glaciale* subsp. *glaciale*, *D. glaciale* subsp. *calcareum* and *D. grandiflorum* were DNA diploids (‘diploids’ hereafter) and all of *D. stiriacum* were DNA tetraploids (‘tetraploids’ hereafter). *Pisum sativum* was used as internal standard for 51 out of 58 populations, whereas *Vicia faba* was used for populations 5, 6, 28, 40, 44, 55 and 57. By mistake, population 28 was not analysed.

**Fig 2:**
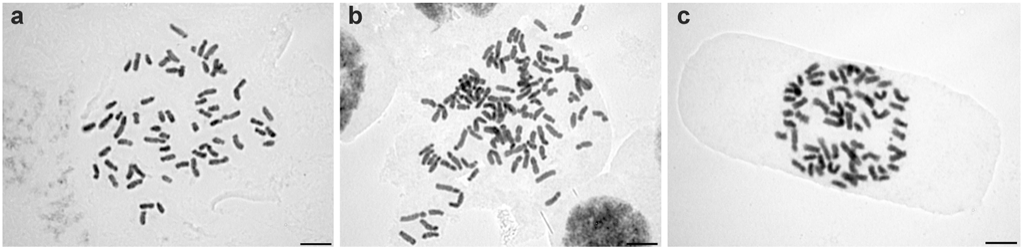
Mitotic chromosomes of members of the *Doronicum clusii* aggregate. (a) *D. clusii* s.s. from Mt. Plose, Dolomiten, Italy (pop. 42; CP1042), 2*n* = 60; (b) *D. stiriacum* from Mt. Höchstein, Schladminger Tauern, Austria (pop. 34; CP1041), 2*n* = c. 120; (c) *D. glaciale* subsp. *glaciale* from Mt. Gruft, Gurktaler Alpen, Austria (pop. 15; CP1038), 2*n* = 60. Scale bars = 5 μm.

Chromosome numbers of diploids and tetraploids were determined to be 2*n* = 2*x* = 60 and 2*n* = 4*x* = c. 120, respectively (Fig. 2). A summary of new and published chromosome numbers is given in Table 4.

**Table 4:** Published and new chromosome counts from the *Doronicum clusii* aggregate and from *D. grandiflorum*.

Population	Taxon	Geographic origin^a^; voucher information	Ploidy	*n*/2*n* (no. of ind. counted)	Reference
6	‘intermediate’ ^b^	A: Grazer Bergland, Hochlantsch; WU: CP1037, http://herbarium.univie.ac.at/database/detail.php?ID=546686	2*x*	2*n* = c. 60 (1)	This study
15	*D. glaciale* subsp. *glaciale*	A: Gurktaler Alpen, Gruft; WU: CP1038, http://herbarium.univie.ac.at/database/detail.php?ID=546696	2*x*	2*n* = 60 (1)	This study
32	*D. stiriacum*	A: Gurktaler Alpen, Kaserhöhe; WU: CP1039, http://herbarium.univie.ac.at/database/detail.php?ID=546719	4*x*	2*n* = c. 120 (1)	This study
34	*D. stiriacum*	A: Schladminger Tauern, Höchstein; WU: CP1040: http://herbarium.univie.ac.at/database/detail.php?ID=550851; CP1041: http://herbarium.univie.ac.at/database/detail.php?ID=548653	4*x*	2*n* = c. 120 (2)	This study
35	*D. stiriacum*	A: Schladminger Tauern, Großes Gurpitscheck, WU: CP1026, http://herbarium.univie.ac.at/database/detail.php?ID=548654	4*x*	2*n* = c. 120 (1)	This study
42	*D. clusii* s.s.	I: Dolomiten, Plose; WU: CP1042, http://herbarium.univie.ac.at/database/detail.php?ID=550250	2*x*	2*n* = 60 (1)	This study
55	*D. grandiflorum*	A: Dachsteinmassiv, Krippenstein; WU: CP1043, http://herbarium.univie.ac.at/database/detail.php?ID=550852	2*x*	2*n* = 60 (1)	This study
	*D. glaciale* subsp. *calcareum* (as *D. calcareum*)	A: Rax-Schneeberg-Gruppe, Schneeberg	2*x*	*n* = 30	[131]
	*D. clusii* s.s.	A: Stubaier Alpen, Windachtal ob Sölden	2*x*	2*n* = 60 ± 1	[132]
	*D. clusii* s.s.	CH: Albula-Alpen, Val Muraigl	2*x*	*n* = 30	[133]
	*D. clusii* s.s.	CH: Walliser Alpen, S of Gamsen near Brig, left side of Nanztal	2*x*	2*n* = 60	[134]
	*D. glaciale* subsp. *glaciale*	SLO: Julijske Alpe, Šija	2*x*	2*n* = 60	[135]
	*D. stiriacum*	SK: Západné Tatry, Baranec and Smutná dolina	4*x*	2*n* = 120	Murín in [136]
	*D. stiriacum* (as *D. clusii*)	UA: Chornohora, Pop Ivan	4*x*	2*n* = 120	[137]
	*D. stiriacum* (as *Aronicum clusii*)	PL: Tatry Wysokie, 3 different localities	4*x*	2*n* = c. 120	Wcisło in [138]
	*D. stiriacum* (as *D. clusii*)	PL: Tatry Wysokie, 16 different localities	4*x*	2*n* = 120	[55]
	*D. grandiflorum*	CH/F: Alpes valaisannes, Col de Balme	2*x*	*n* = 30	[139]
	*D. grandiflorum*	F: Pyrénées centrales, Llaurenti [Laurenti]	2*x*	*n* = 30	[140]
	*D. grandiflorum*	F: Pyrénées orientales, Pic Peric	2*x*	*n* = 30	[140]

^a^ countries: A, Austria; CH, Switzerland; F, France; I, Italy; SK, Slovakia; SLO, Slovenia; UA, Ukraine.

^b^ morphological intermediates between *D. glaciale* subsp. *calcareum* and *D. glaciale* subsp. *glaciale*.

### AFLPs

After removing one monomorphic fragment, 47 fragments present in all but one individual as well as 41 non-reproducible fragments, the final data matrix (available from Dryad under http://dx.doi.org/10.5061/dryad.573pm) contained 319 polymorphic fragments in 276 individuals. In the AFLP profiles from replicated samples 949 differences were observed out of 27,744 phenotypic comparisons, resulting in an error rate of 3.42%.

**Fig 3:**
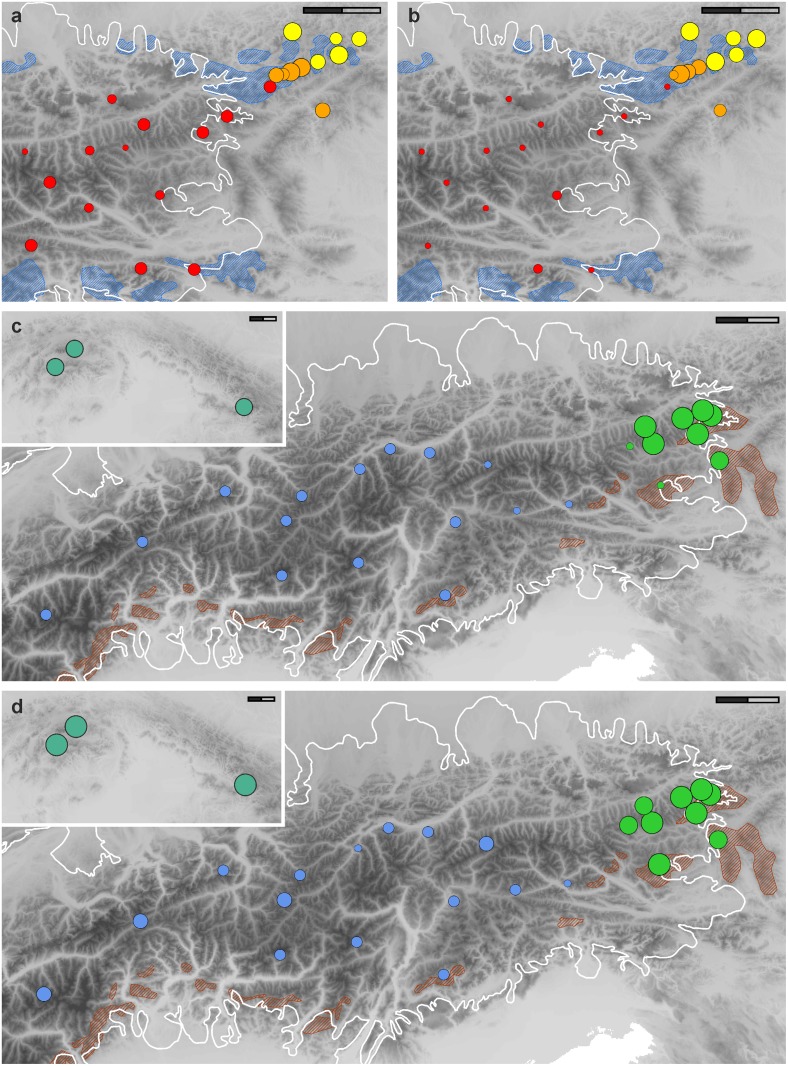
Patterns of (a, c) AFLP diversity and (b, d) rarity within populations of the *D. clusii* aggregate. (a, b) *D. glaciale* subsp. *calcareum* (yellow), *D. glaciale* subsp. *glaciale* (red) and their morphological intermediates (orange); (c, d) *D. clusii* s.s. (blue) and *D. stiriacum* (green) from the Alps and the Carpathians (insert). Dot sizes are proportional to genetic diversities and rarities given in Table 2; five weighted categories were used. The maximum extent of the Alpine ice shield during the Last Glacial Maximum (LGM) is given as white line; hatched areas in blue and red indicate glacial refugia (i.e., areas outside the continuous glaciation during the LGM and areas within the ice shield but situated below the LGM snow line) on calcareous and siliceous bedrock, respectively (modified from [5]); the Carpathians were only locally glaciated, not exceeding 1% of their total area [125]. Scale bars: 50 km.

Genetic diversity (Table 2, Fig. 3) varied approximately 22-fold among populations ranging from 0.006 in population 41 (*D. clusii* s.s.) to 0.137 in population 8 (morphological intermediate between *D. glaciale* subsp. *calcareum* and *D. glaciale* subsp. *glaciale*); the highest value in a non-admixed population was 0.134 in population 29 (*D. stiriacum*). Rarity varied sevenfold ranging from 0.48 in population 26 (*D. × bauhini*) to 3.20 in population 38 (*D. stiriacum*; Table 2, Fig. 3). After removing *D. × bauhini* and morphological intermediates between *D. glaciale* subsp. *glaciale* and *D. glaciale* subsp. *calcareum*, both *D. clusii* s.s. and *D. glaciale* subsp. *glaciale*, two taxa that occur mainly in formerly glaciated areas, exhibited significantly lower global genetic diversity and rarity (Tukey—Kramer test, α = 0.05) than both *D. glaciale* subsp. *calcareum and D. stiriacum* (Table 2, Fig. 3), two taxa that mainly occur in formerly unglaciated (refugial) areas. Alpine populations of *D. stiriacum* from refugia (populations 27–30, 32, 36–38) and from glaciated areas (populations 31, 33–35) did not differ with respect to genetic diversity (two-tailed *t*-test, *t* = 1.710, df = 10, *P* = 0.868) and rarity (two-tailed *t*-test, *t* = 1.537, df = 10, *P* = 0.155). Means of genetic diversity and rarity in *D. glaciale* subsp. *calcareum* and *D. glaciale* subsp. *glaciale* (excluding the morphologically intermediate populations 6–10) were significantly different (diversity: two-tailed *t*-test, *t* = 3.309, df = 17, *P* = 0.004; rarity: Mann—Whitney *U*-test, two tailed significance: *Z* = -3.256, *P* < 0.001). The number of private markers (i.e., restricted to a single taxon) was zero in *D. glaciale* subsp. *glaciale*, six in *D. glaciale* subsp. *calcareum*, 20 in *D. clusii* s.s., and 50 in *D. stiriacum*.

**Fig 4:**
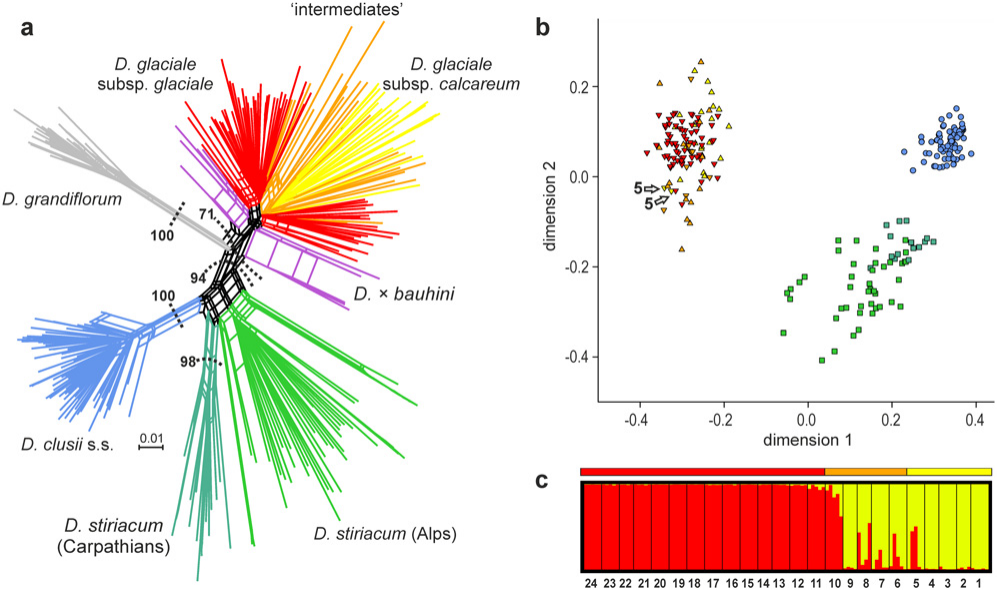
Structure of AFLP variation in the *Doronicum clusii* aggregate. (a) NeighborNet of the complete AFLP data set including the outgroup *D. grandiflorum*; splits with weight < 0.001 were omitted to aid legibility; numbers represent bootstrap values above 50% given for major groups only. (b) Non-metric multidimensional scaling of Jaccard distances using Gaussian clustering with Prabclus; only two of three dimensions are shown. The symbols represent Prabclus-groups (circles: *D. clusii* s.s.; squares: *D. stiriacum*; triangles pointing upwards: *D. glaciale* subsp. *calcareum*; triangles pointing downwards: *D. glaciale* subsp. *glaciale*; *D. × bauhini* was not included), the colours denote taxa as in Fig. 1; the arrows indicate two individuals of *D. glaciale* subsp. *calcareum* from population 5 that are assigned to *D. glaciale* subsp. *glaciale*. (c) Estimated population structure among *D. glaciale* subsp. *calcareum* (pops. 1–5), *D. glaciale* subsp. *glaciale* (pops. 11–24) and morphological intermediates (pops. 6–10) inferred from Bayesian clustering with STRUCTURE at K = 2. Each individual is represented by a vertical bar, black lines separate populations, and population numbers (as in Table 2) are given from west to east; the coloured bar above the barplots represents morphologically defined taxa as in Fig. 1.

The NeighbourNet (Fig. 4) showed that the *D. clusii* aggregate (bootstrap support BS 100) comprises three groups corresponding to *D. clusii* s.s. (BS 100), *D. stiriacum* (BS <50) and *D. glaciale* subsp. *glaciale* plus *D. glaciale* subsp. *calcareum* (BS 71). Gaussian clustering yielded four groups corresponding to *D. clusii* s.s., *D. stiriacum*, *D. glaciale* subsp. *glaciale* and *D. glaciale* subsp. *calcareum* (Table 2). Plotting of NMDS data showed three clearly distinguishable groups (Fig. 4); a weak separation of *D. glaciale* subsp. *calcareum* from *D. glaciale* subsp. *glaciale* was only evident along the third axis (not shown). Two individuals of population 5 of *D. glaciale* subsp. *calcareum* were mis-assigned to *D. glaciale* subsp. *glaciale* (Table 2, Fig. 4). Morphologically intermediate populations mostly grouped with *D. glaciale* subsp. *calcareum* (15 individuals: all from populations 7 and 9 and four and two from populations 6 and 8, respectively) whereas all individuals from the westernmost population 10 and the remaining individuals from populations 6 and 8 were assigned to *D. glaciale* subsp. *glaciale* (Table 2). The assignment of *D. clusii* s.s. and *D. stiriacum* to the respective groups was stable with high proportions of correct assignment (0.82–1.00 in *D. clusii* s.s. and 0.56–0.91 in *D. stiriacum*) and a negligible proportion of incorrect assignment (a single individual in a single bootstrap replicate was mis-assigned to *D. clusii* s.s.). The assignment of *D. glaciale* subsp. *calcareum* and *D. glaciale* subsp. *glaciale* to the respective clusters was less stable because the proportion of incorrect assignment of *D. glaciale* subsp. *glaciale* to the *D. glaciale* subsp. *calcareum* group was 0.23–0.39. Placement of the two mis-assigned individuals from population 5 (*D. glaciale* subsp. *calcareum*) was not stable, because the genetic assignment (to *D. glaciale* subsp. *glaciale*) was only 0.53–0.56 compared to the taxonomic assignment (to *D. glaciale* subsp. *calcareum*) of 0.29–0.30. The assignment of individuals from morphologically intermediate populations was not stable, either, and the proportion of correct assignment to the *D. glaciale* subsp. *calcareum* or *D. glaciale* subsp. *glaciale* group was 0.42–0.93 and 0.32–0.73, respectively.

Among *D. glaciale* subsp. *calcareum*, *D. glaciale* subsp. *glaciale* and their morphological intermediates, STRUCTURE distinguished two groups (Fig. 4). The two groups corresponded to populations 1–4 of *D. glaciale* subsp. *calcareum* plus population 9 (morphological intermediate with *D. glaciale* subsp. *glaciale*) and populations 11–24 of *D. glaciale* subsp. *glaciale*, respectively; the remaining morphologically intermediate populations were genetically admixed with either the *D. glaciale* subsp. *calcareum* gene pool (populations 5–8) or the *D. glaciale* subsp. *glaciale* gene pool (population 10) dominating.

Among *D. clusii* s.s., *D. glaciale* subsp. *glaciale* and their putative hybrid (*D. × bauhini*), STRUCTURE identified two groups. Although *D. × bauhini* was genomically admixed, alleles from *D. glaciale* subsp. *glaciale* dominated (at least 73% relative contribution in population 25 and at least 98% in population 26).

### DNA sequences

**Fig 5:**
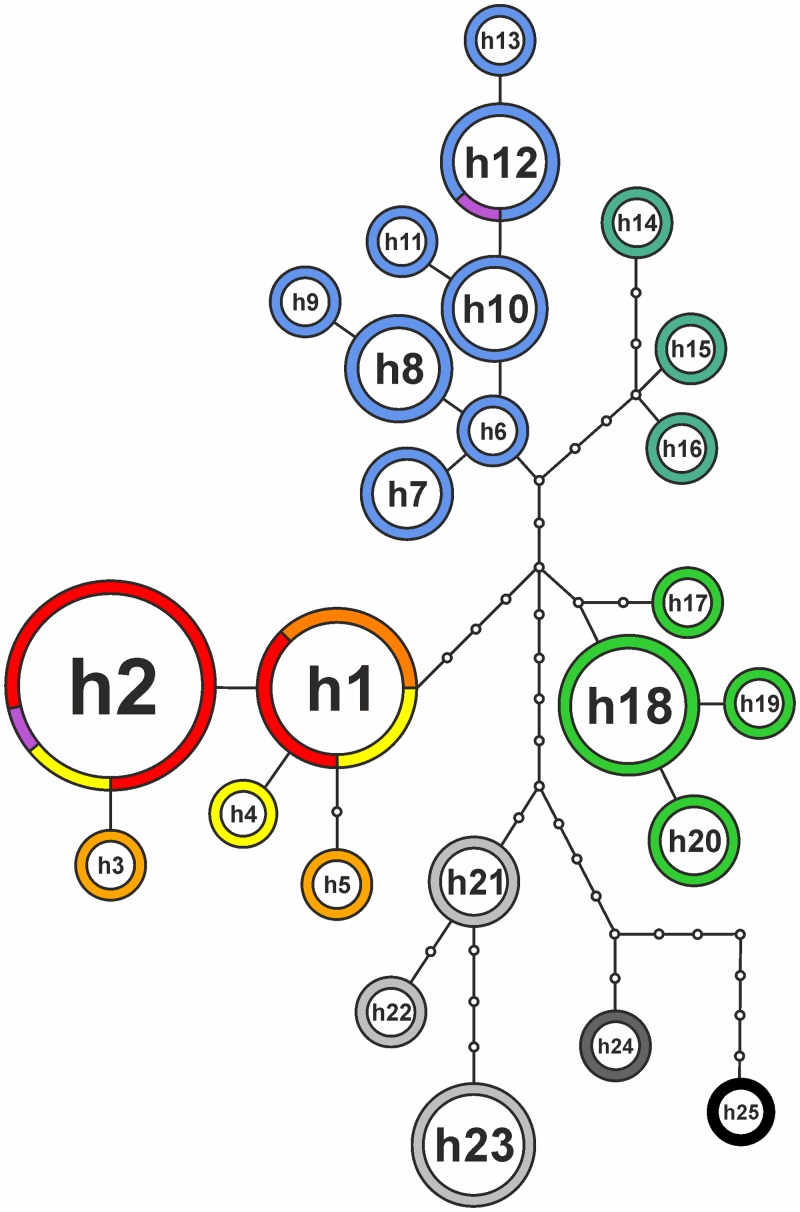
Phylogenetic relationships of the *Doronicum clusii* aggregate based on plastid DNA sequences. Statistical parsimony network of plastid haplotypes; non-sampled haplotypes are shown as small open circles. The diameter of circles is proportional to the number of sampled haplotypes. Colour-coding of taxa as in Fig. 1; outgroup species: *D. grandiflorum* (light grey), *D. carpaticum* (middle grey), *D. austriacum* (black).

Sequences are available from GenBank (see Table 2 for accession numbers). Sequence alignments are available from Dryad under http://dx.doi.org/10.5061/dryad.573pm. Sequence statistics are given in Table 5. Apart from excluded mononucleotide repeats, chloroplast markers showed no intra-populational variation. The statistical parsimony network, comprising 25 haplotypes from 62 sequences (Table 2, Fig. 5), shows that apart from the clearly separated outgroup species *D. austriacum*, *D. carpaticum* and *D. grandiflorum*, four haplotype groups are evident corresponding to *D. clusii* s.s., *D. glaciale* subsp. *glaciale* plus *D. glaciale* subsp. *calcareum*, *D. stiriacum* from the Carpathians and *D. stiriacum* from the Alps. Haplotype 12 of *D. clusii* s.s., which is common in the Central Alps, is shared by population 25 of *D. × bauhini*, whereas haplotype 2, the most common haplotype of *D. glaciale* subsp. *glaciale* plus *D. glaciale* subsp. *calcareum*, is found in population 26 of *D. × bauhini*.

**Table 5:** Sequence statistics of studied DNA regions.

Markers	Sequence length (bp)	Alignment length (bp)	Variable characters^a^	Parsimony-informative characters^a^
*ndh*F-*rpl*32	1052–1082	1099	14/7	9/5
*rpl*32-*trn*L_UAG_	857–860	860	13/7	10/5
3′*rps*16–5′*trn*K_UUU_	858–879	924	14/10	6/4
combined plastid regions	2769–2805	2883	41/24	26/15
combined plastid regions recoded	2763–2765	2776	41/24	25/14
ITS	726–730	731	161/136	89/84

^a^ with / without outgroup sequences.

**Fig 6:**
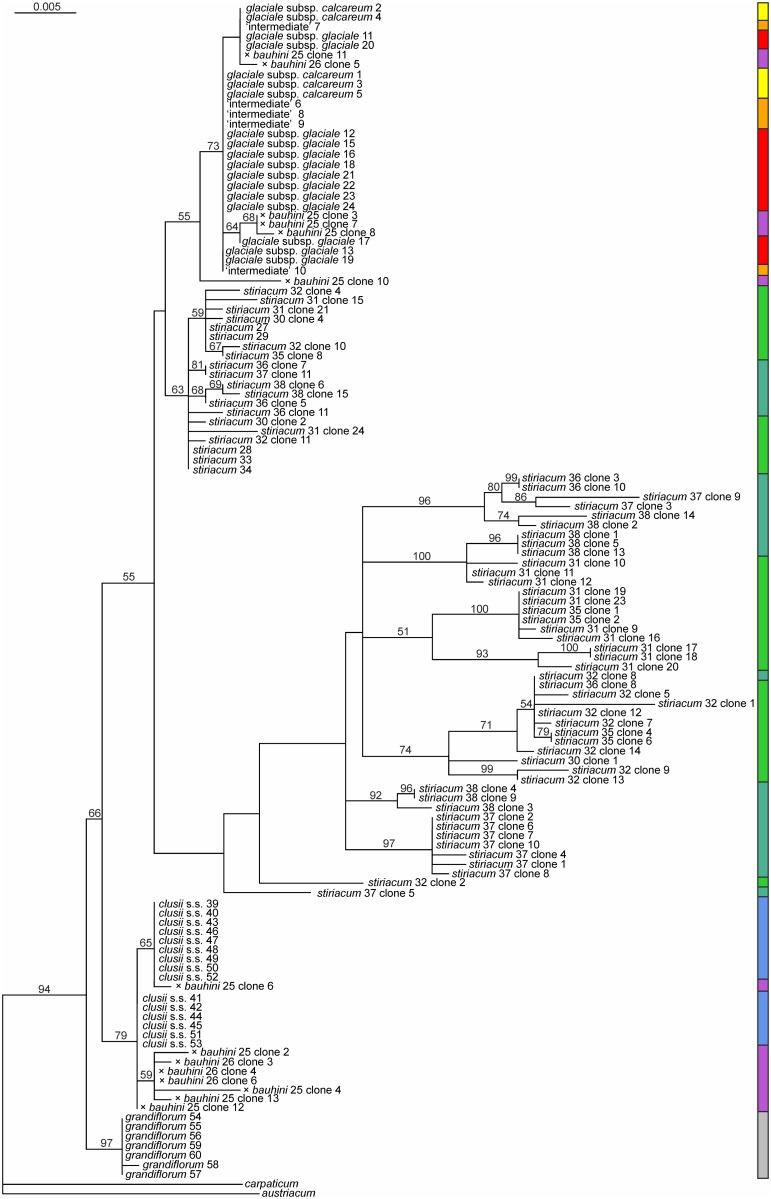
Phylogenetic relationships of the *Doronicum clusii* aggregate based on nuclear DNA sequences. Maximum likelihood tree based on nuclear ITS data; numbers above branches are bootstrap support values above 50%. The coloured bar to the right represents morphologically defined taxa as in Fig. 1.

In the maximum likelihood tree of the 127 ITS sequences (-ln 2567.7; Fig. 6) the *D. clusii* aggregate forms a monophyletic group (BS 66). *Doronicum clusii* s.s., which shows no intraspecific sequence variation beyond a few ambiguous sites (Table 3), groups with cloned sequences of both *D. × bauhini* populations (BS 79). *Doronicum stiriacum* does not form a monophyletic group, but falls into two clades. An unsupported clade containing cloned sequences from the Carpathians (populations 36–38) and some, but not all populations from the Alps (populations 30–32, 35) is weakly supported sister (BS 56) to the unsupported clade containing *D. glaciale* subsp. *calcareum*, *D. glaciale* subsp. *glaciale* and *D. stiriacum*. Sequences of *D. stiriacum* (from all populations) form a clade (BS 63) sister group to a clade (BS 55) comprising *D. glaciale* subsp. *calcareum*, *D. glaciale* subsp. *glaciale*, their morphological intermediates and cloned sequences of both *D. × bauhini* populations. Samples from *D. glaciale* subsp. *calcareum*, *D. glaciale* subsp. *glaciale* and their morphological intermediates intermix, and phylogenetic structure, if present, does not correspond to taxonomic boundaries.

## Discussion

### Phylogeny and taxonomy of the *D. clusii* aggregate

The *D. clusii* aggregate is confirmed as a cohesive group distinct from *D. grandiflorum* (Figs. 5, 6). This group comprises three genetic lineages (Figs. 4–5), which correspond to diploid *D. clusii* s.s., tetraploid *D. stiriacum* and diploid *D. glaciale* subsp. *glaciale* plus *D. glaciale* subsp. *calcareum* (referred to as *D. glaciale* s.l. in the following). Álvarez Fernández et al. [49] did neither include *D. glaciale* subsp. *calcareum* nor *D. stiriacum*, which were considered conspecific with *D. glaciale* subsp. *glaciale* and *D. clusii* s.s., respectively [50], and ours are the first molecular results on both taxa. Due to overall morphological similarity *D. stiriacum* has often been treated as subspecies of *D. clusii* s.s. (e.g., [113]) or as a synonym of *D. clusii* s.s. (e.g., [50]). As shown here (Figs. 4–6), *D. stiriacum* is genetically clearly separated from *D. clusii* s.s. and, therefore, should be treated at the species level. This is also justified by differences in morphology (thicker, coarse leaves that are densely villous on both sides, sparse glands on the involucrum, and villous corolla tubes in *D. stiriacum* versus tender leaves that are almost glabrous on the upper side, abundant glands on the involucrum and scape, and glabrous corolla tubes in *D. clusii* s.s.; S1 Appendix), ploidy level (tetraploid versus diploid) and distribution range (eastern-most Alps and Carpathians versus Alps except for the eastern-most parts [59,114]).

The regionally endemic *D. glaciale* subsp. *calcareum* is weakly, but consistently differentiated from widespread *D. glaciale* subsp. *glaciale*. Plastid DNA haplotypes found in *D. glaciale* subsp. *calcareum* are shared with *D. glaciale* subsp. *glaciale* (Fig. 5) and ITS shows no divergence between the two taxa (Fig. 6). Except for the NeighborNet analysis (Fig. 4A), AFLP data are structured according to the morphological differentiation of *D. glaciale* subsp. *calcareum* and *D. glaciale* subsp. *glaciale* (Fig. 4B, C). This pattern is only blurred by population 5, which is genetically admixed and contains individuals mis-assigned by Gaussian clustering (Fig. 4B), but is not morphologically intermediate. The distinction between *D. glaciale* subsp. *calcareum* and *D. glaciale* subsp. *glaciale* implied by Gaussian clustering is, however, not stable with an average bootstrap support of only 72 for an individual’s correct assignment. As expected, morphological intermediates (pops. 6–10) show varying levels of AFLP admixture (Fig. 4C) and, consequently, are assigned to either *D. glaciale* subsp. *calcareum* or *D. glaciale* subsp. *glaciale* (Fig. 4B). As *D. glaciale* subsp. *calcareum* and *D. glaciale* subsp. *glaciale* are genetically connected in a wide contact zone mainly containing morphological intermediates, have parapatric distribution ranges (Fig. 1) and show only subtle differences in leaf and involucrum indumentum (S1 Appendix), we follow recommendations by [115] for sexually reproducing flowering plants and retain the subspecific rank for the taxon from the northeastern-most Alps originally described as separate species, *D. calcareum* Vierh. [58].

Our data confirm the status of *D. × bauhini* as hybrid between *D. clusii* s.s. and *D. glaciale* subsp. *glaciale*, as suggested previously based on morphology [48,51]. Based on cloned ITS sequences, the two populations of *D. × bauhini* group with both *D. clusii* s.s. and *D. glaciale* s.l. (Fig. 6). Similarly, population 25 possesses haplotype 12 common in *D. clusii* s.s., whereas population 26 possesses haplotype 2 common in *D. glaciale* s.l. (Fig. 5, Table 2). This suggests that both *D. clusii* s.s. and *D. glaciale* (based on geographical considerations almost certainly subsp. *glaciale*) acted as maternal parents. In the NeighborNet of AFLP-data *D. × bauhini* populations are closer to *D. glaciale* s.l. than to *D. clusii* s.s. (Fig. 4B), which agrees with the higher proportion of the *D. glaciale* s.l. genome in *D. × bauhini* inferred from STRUCTURE analyses. This likely is due to backcrossing and corresponds well to a higher morphological affinity to *D. glaciale* subsp. *glaciale*. Higher morphological similarity to one parent was also found in *D*. × *minutilloi* Peruzzi (*D. columnae* Ten. × *D. orientale* Hoffm.) from Central Italy [52]. Backcrossing is feasible, because *D. × bauhini* possesses regularly developed pollen as confirmed by SEM analysis (pollen from population 25; H. Halbritter and C. Pachschwöll, unpubl. data).

### Origin of polyploid *D. stiriacum*

Flow cytometry and chromosome counts revealed constant tetraploidy of *D. stiriacum* over the entire distribution range including the hitherto not investigated Alps (Tables 2, 4). The molecular data indicate that tetraploid *D. stiriacum* is a distinct evolutionary lineage clearly separated from diploid *D. clusii* s.s. and *D. glaciale* s.l. and exhibiting a higher number of private AFLP fragments than any other member of the *D. clusii* aggregate. Molecular evidence is, however, inconclusive with respect to an auto- or allopolyploid origin. The mode of polyploidization (auto‐ versus allopolyploidy) was not discussed by the authors studying karyology and morphology of *D. stiriacum* (e.g., [55,114]). The hypothesis of an autopolyploid origin from *D. clusii* s.s. (or an extinct diploid lineage) finds support in overall morphological and ecological similarities between the two species [59,114] and the lack of signal for mixed ancestry in the sequence data (Figs. 5, 6). The alternative hypothesis of an allopolyploid origin involving *D. glaciale* s.l. and *D. clusii* s.s. has not been considered previously, but would be in line with morphological traits resembling *D. glaciale* s.l., such as the thick, coarse leaves and the rare presence of stipitate glands on the basal leaves [59,114,116], as well as the presence of hybrids in contact areas (i.e., *D. × bauhini*). Further evidence comes from the lack of monophyly of *D. stiriacum* in the ITS phylogeny (Fig. 6) that may, however, be due to divergent evolution of different 35S rDNA loci [64], the intermediate position between *D. clusii* s.s. and *D. glaciale* s.l. in the AFLP NeighborNet and the lack of long splits supporting *D. stiriacum* (Fig. 4A), a pattern resembling the recently evolved allopolyploid *Androsace brigantiaca* Jord. & Fourr. [117].

### Phylogeography and spatiotemporal evolution

Species responded idiosyncratically to Pleistocene climatic oscillations [118], but generally higher values of genetic rarity—but not necessarily of genetic diversity [119]—are expected in refugia or rear edge populations than in recolonized areas [2,39,42,120,121]. Impact of climatic oscillations, likely during the Pleistocene, on genetic patterns is evident in the *D. clusii* aggregate and can be best seen in the elevated levels of genetic diversity and rarity (Fig. 3) in *D. glaciale* subsp. *calcareum* and *D. stiriacum*, whose ranges are either almost entirely restricted to peripheral refugia in the northeastern Alps or only slightly extend westwards from refugia in the easternmost Central Alps [5]. In contrast, *D. clusii* s.s. and *D. glaciale* subsp. *glaciale* are widely distributed mostly in previously strongly glaciated areas and possess significantly lower global genetic diversity and rarity (Fig. 3).

At the eastern margin of the distribution range of *D. clusii* s.s., where this species is rare and restricted to a few isolated populations (populations 39–41), genetic diversity is low. Population 39 possesses the lowest rarity of all populations (Fig. 3, Table 2) and is divergent in the NeighborNet (Fig. 4), most probably due to its genetic depauperation. Each of populations 39–41 has a unique haplotype (9, 11, 13; Fig. 5) suggesting eastwards expansion, which stopped in the Hohe Tauern (part of the Central Alps: Fig. 1), a frequently emerging break zone of alleles and species distributions in silicicolous species [22]. Otherwise, no phylogeographic structure can be discerned neither by AFLP variation nor by the uniform ITS sequences (Fig. 6). As our study focused on the eastern Alps as the only area of co-occurrence of taxa of the *D. clusii* aggregate, the southwestern and the middle southern Alps, which may have acted as important glacial refugia [5] also for *D. clusii* s.s., have remained unsampled or are underrepresented.

Vierhapper [58] postulated a recent, postglacial origin for *D. stiriacum*, which he deemed responsible for its restriction to a small area. However, the divergence between Alpine and Carpathian populations in all genetic data (Figs. 4–6) and the possession of plastid haplotypes not found in the diploid species (Fig. 5) can be better explained by an earlier preglacial origin of *D. stiriacum*. An earlier origin is supported by macrofossils from the northern foothills of the western Carpathians with an estimated age of c. 29,500 cal years BP [121] thus predating the Last Glacial Maximum in the western Carpathians [122–124]. This fits well to the hypothesized downward displacement of alpine vegetation and is in line with the suggested predominance of herbaceous arctic‐alpine species in the Carpathian forelands during that period [125]. A plausible scenario for the evolution of *D. stiriacum* includes an eastern Alpine origin (based on the distribution of the species’ closest relatives) and subsequent dispersal to the Carpathians, a scenario suggested for other silicicolous species such as *Jacobaea carniolica* (Willd.) Schrank (syn. *Senecio carniolicus* Willd.; [76]), *Salix herbacea* L. [126] or *Cardamine resedifolia* L. [127]. Subsequently, divergence took place in phases of vicariance and glacial survival in both the Alps and Carpathians. As no difference in genetic rarity emerged between refugia (populations 27–30, 32, 36–38) and glaciated areas (populations 31, 33–35; Fig. 3) in the Alps, slow, broad-fronted diffusion likely led to the small, but compact current distribution. For *D. stiriacum* a pronounced post-glacial migration lag resulting in incomplete range filling in the eastern Alps was modelled [12], suggesting that the species’ current distribution range can be better explained with historical than with ecological factors. This strong migration lag and the limited extent of postglacial range expansion may also explain the lack of strong imprints of refugial patterns as observed in other silicicolous species of similar distribution [127–129].

*Doronicum glaciale* s.l. exhibits a particularly clear imprint of its glacial history. *Doronicum glaciale* subsp. *calcareum* from the previously only locally glaciated northeastern Alps exhibits high levels of AFLP diversity and rarity (Fig. 3, Table 2), whereas *D. glaciale* subsp. *glaciale*, predominantly distributed in formerly glaciated areas, is strongly depauperate (Fig. 3). In none of the hitherto investigated calcicolous Alpine species [16,63,82,90] were such strong intraspecific patterns observed. A likely hypothesis is that westward leading-edge colonization [120] started from source populations on the Hochschwab Massif (the area, where the morphological intermediate populations 7–10 occur: Fig. 1), which are genetically intermediate (Fig. 4) and possess short glands on the basal leaves, a morphological feature not found in eastern populations of *D. glaciale* subsp. *calcareum* (S1 Appendix). This hypothesis identifies the Hochschwab as primary contact zone and suggests parapatric origin of *D. glaciale* subsp. *glaciale* from *D. glaciale* subsp. *calcareum*. The derived *D. glaciale* subsp. *glaciale* exhibits a wider niche (extension onto basic silicates) as observed in South American *Pozoa volcanica* Mathias & Constance [130]. Alternatively, *D. glaciale* subsp. *glaciale* might have undergone a founder effect within the unglaciated easternmost Central Alps, from where it subsequently expanded its range also north(east)wards to finally form a secondary contact zone at the Hochschwab Massif. This hypothesis is, however, less likely as populations in the contact zone do not show increased genetic diversity (Fig. 3, Table 2) as expected for an evolutionary melting pot [119].

## Conclusion

Our study identifies range shifts, which were likely triggered by Pleistocene climatic oscillations, and polyploidisation as main forces shaping the evolutionary history and consequently the genetic structure of *D. clusii* and relatives. Integrating morphological, karyological, genetic and biogeographic data allows us to draw solid taxonomic conclusions with respect to controversial taxa (*D. stiriacum*, *D. glaciale* subsp. *calcareum*). The highly uneven distribution of genetic variation across species strongly suggests that a better understanding of the evolution of intricate alpine species groups is necessary for designing conservation strategies for endemics in the light of global warming.

## Supporting Information

S1 Appendix Systematics, morphological differentiation, ecology and geographical distribution of the constituents of the *Doronicum clusii* aggregate and its closest relative *D. grandiflorum*. (PDF)
