# Supplementary material for: Polyploidisation and Geographic Differentiation Drive Diversification in a European High Mountain Plant Group (Doronicum clusii Aggregate, Asteraceae)
Source: PLoS One. 2015 Mar 6;10(3):e0118197. doi: 10.1371/journal.pone.0118197 (PMC4352020; doi:10.1371/journal.pone.0118197)
Supplement: S1 Appendix — (PDF) [file pone.0118197.s001.pdf]

S1 Appendix: Systematics, morphological differentiation, ecology and geographical distribution of the constituents of the *Doronicum clusii* aggregate and its closest relative *D. grandiflorum*.

***D. glaciale* (Wulf.) Nyman subsp. *calcareum* (Vierh.) Hegi (=*Doronicum calcareum* Vierh., *D. glaciale* (Wulf.) Nyman var. *calcareum* (Vierh.) Cavill.)**

Doronicum glaciale subsp. *calcareum* was originally described by Vierhapper [1] on the specific level and later treated as a species [2,3], variety [4], or as today, as subspecies [5–7]. Álvarez Fernández and Nieto Feliner [8], however, stated that 'var. *calcareum* is of no taxonomic value' and did not include this taxon in their studies [9,10]. Together with about 30 angiosperms such as *Achillea clusiana* Tausch (Asteraceae), *Alyssum neglectum* Magauer, Frajman & Schönsw. (Brassicaceae), *Dianthus alpinus* L. (Caryophyllaceae), *Noccaea crantzii* F.K.Mey. (= *Thlaspi alpestre* Crantz; Brassicaceae), *Scorzoneroides montaniformis* (Widder) Gutermann (= *Leontodon montaniformis* Widder; Asteraceae) or *Primula clusiana* Tausch (Primulaceae), it is endemic to the northeastern-most Alps in Austria [7,11–14], an area known for a high degree of endemism due to its limited ice cover during Pleistocene glaciations [15,16]. The distribution of *D. glaciale* subsp. *calcareum* ranges from Mt. Schneeberg in the east to the Hochschwab Massif in the west (Fig. A). It is restricted to purely calcareous bedrock and centered in snowbed communities (Arabidion caeruleae) but can also grow in alpine meadows or stabilized screes [17].

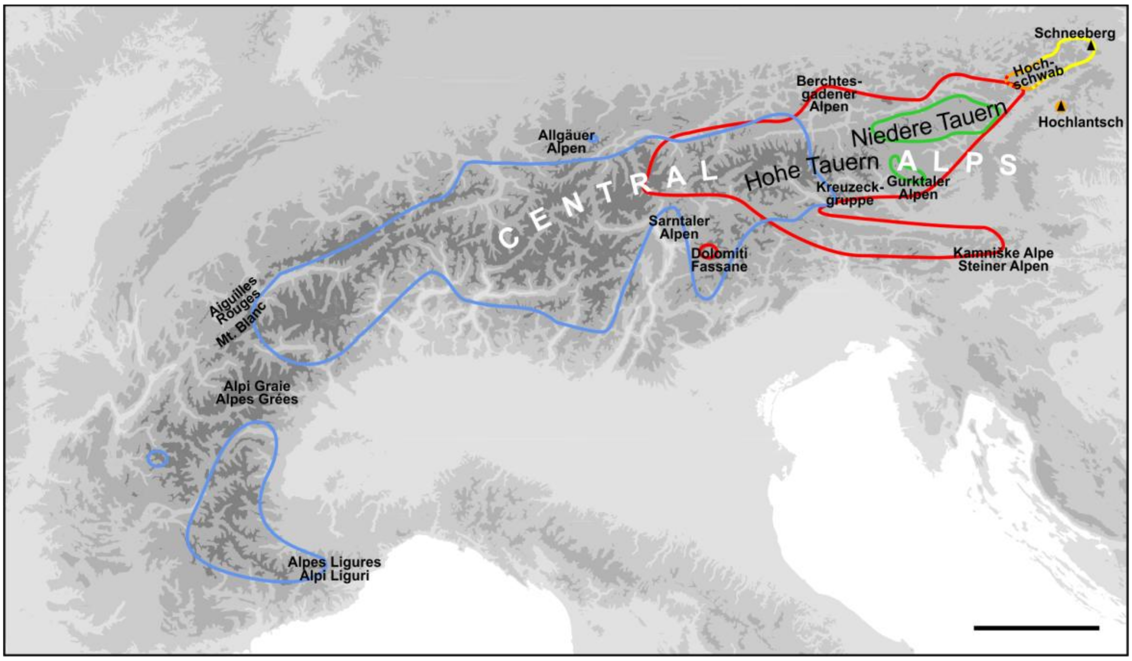

Figure A. Distribution of members of the *D. clusii* aggregate. Species are colour-coded: *D. glaciale* subsp. *calcareum* (yellow), *D. glaciale* subsp. *glaciale* (red) and their morphological intermediates (orange); *D. stiriacum* (green); *D. clusii* s.s. (blue). Scale bar = 100 km.

Doronicum glaciale* (Wulf.) Nyman subsp *glaciale

Doronicum glaciale s.s. was described from the Hohe Tauern, Central Alps, Austria [8,18]. This Alpine endemic is distributed throughout much of the eastern Alps, from the western Hochschwab Massif (Austria) in the east to the Sarntaler Alpen (Italy) in the west and from the Berchtesgadener Alpen (Germany) in the north to the Kamniške Alpe/Steiner Alpen (Slovenia/Austria) in the southeast (Fig. A; [18,19]). Isolated occurrences are known from the Dolomiti Fassane, Italy (Fig. A; [10,20]). It has a wider ecology than *D. glaciale* subsp. *calcareum* and occurs on limestone and on basic, intermediate to slightly acidic substrate like schists in open rocky places, screes and moraines (Androsacion alpinae, Drabion hoppeanae and Thlaspion rotundifolii; [18]).

***Doronicum glaciale* subsp. *glaciale* and *D. glaciale* subsp. *calcareum*: their morphological distinction and putative hybrid zone**

The morphological differentiation between *D. glaciale* subsp. *calcareum* and *D. glaciale* subsp. *glaciale* is fairly weak and is based on hair characters only [5,7], leading Álvarez Fernández and Nieto Feliner [8] and Álvarez Fernández [10] to reject *D. glaciale* subsp. *calcareum* as a separate taxon. Whereas the basal leaves of *D. glaciale* subsp. *glaciale* possess abundant glandular stipitate hairs, such glands are lacking in *D. glaciale* subsp. *calcareum* (Fig. B; [1,7]). Furthermore, the involucral bracts of *D. glaciale* subsp. *calcareum* bear 0.3–0.5 mm long glandular hairs and 1–2 mm long eglandular hairs, whereas *D. glaciale* subsp. *glaciale* has only shortly stipitate to sessile glands and 0.5–1 mm long eglandular hairs [7]. In between the main distribution ranges of both taxa, morphologically intermediate populations occur in a c. 20 km wide contact zone on the Hochschwab Massif and, geographically isolated, on Palaeozoic limestone on Mt. Hochlantsch (Grazer Bergland, eastern-most Central Alps; Fig. A). Individuals from these populations possess short glands on the basal leaves and long eglandular hairs on the involucrum [1,4,7].

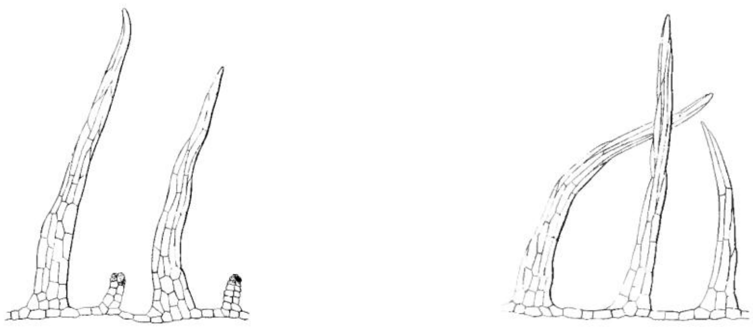

Figure B. Indumentum of basal leaves of *D. glaciale* subsp. *glaciale* (left) and of *D. glaciale* subsp. *calcareum* (right). Modified from [1]. Content provided by the Biodiversity Heritage Library (material not in copyright).

***Doronicum clusii* (All.) Tausch s.s. (= '*D. glabratum*')**

Doronicum clusii s.s. is a western and central Alpine species distributed from the Alpes Ligures/Alpi Liguri (France/Italy) to the Kreuzeckgruppe, Hohe Tauern (Austria), exhibiting a distribution gap in the western parts of the Alpi Graie/Alpes Grées and the Mont Blanc and Aiguilles Rouges Massifs (Fig. A; [21]). One isolated occurrence is known from the Allgäuer Alpen, Austria (Fig. A; [22]). On its easternmost distribution margin in the Hohe Tauern it is rare [21]. *Doronicum clusii* s.s. is an alpine to subnival acidophilic species that tolerates slightly base-rich soils, but does not grow on carbonate bedrock (C. Pachschwöll, pers. obs.). It ascends up to 3,500 m, growing in (pioneer) grassland, moraines, screes and steep slopes centred in the Androsacion alpinae [6,21,23]. *Doronicum clusii* has thin, tender leaves which are almost glabrous on the upper side, abundant glands on involucrum and scape (Fig. C) and glabrous corolla tubes [21].

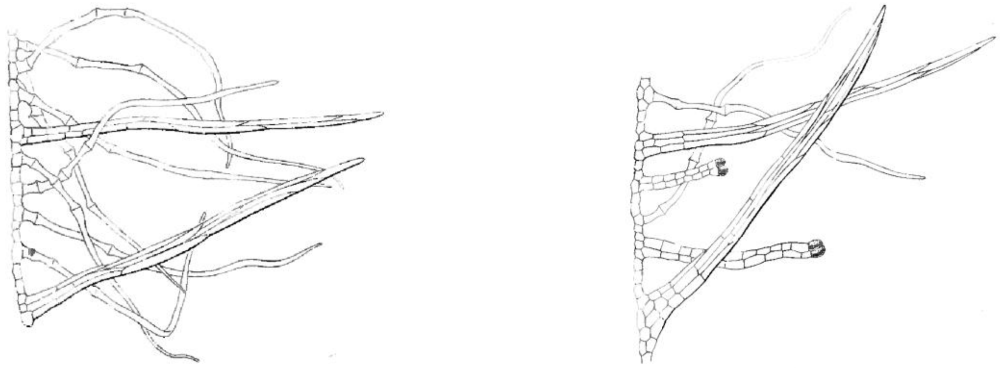

Figure C. Hair morphology of *Doronicum clusii* s.s. and *D. stiriacum* on the leaf margin of basal and cauline leaves (left) and on the margin of involucral bracts (right). Modified from [1]. Content provided by the [Biodiversity Heritage Library](#) (material not in copyright).

***Doronicum stiriacum* (Vill.) Dalla Torre (= *D. clusii* (Vill.) Dalla Torre subsp. *villosum* (Beck) Vierh., '*D. villosum*')**

Doronicum stiriacum and *D. clusii* s.s. are sometimes not distinguished (e.g., [10]), but their separation appears justified based on morphological differences, non-overlapping distribution ranges and different ploidy levels [21,24]. Compared to *D. clusii* s.s., *D. stiriacum* has thicker, coarse and on both sides densely villous leaves, sparse glands on the involucrum and villous corolla tubes [21]. *Doronicum stiriacum* is separated from *D. clusii* s.s. by a distribution gap of c. 70 km (Fig. A) harbouring potentially suitable habitats for both species. The distribution range of *D. stiriacum* is disjunct, ranging from the eastern Central Alps of Austria (Gurktaler Alpen and Niedere Tauern; Fig. A) to the western and eastern Carpathians in Slovakia, Poland, Ukraine and Romania [21]. Indications for the western Central Alps and the western Alps (e.g., [4,11,25]) are erroneous [21]. *Doronicum stiriacum* has very similar ecological requirements as *D. clusii* s.s. It should be noted, that the name *D.*

clusii var. *villosum* Tausch was not validly published [26] although it is frequently used in the literature (e.g., [2,5,6]).

Doronicum* × *bauhini* Saut. (= *D. glaciale* (Wulf.) Nyman f. *bauhini* (Saut.) Dalla Torre & Sarnth.): *D. clusii* s.s. × *D. glaciale* subsp. *glaciale

Putative hybrids are known to link the diploid species *D. clusii* s.s. and *D. glaciale* subsp. *glaciale*. Morphologically, they resemble more closely *D. glaciale* subsp. *glaciale* in possessing many stipitate glands and only a few villous hairs on the basal leaves. Such hybrids were only recorded from areas of overlap of the parental species' distribution ranges in the Central Alps, where they grow on intermediate bedrock such as slightly basic schists [1,20,27–30]. The hybrid status of *D.* × *bauhini* is, however, controversial. It was accepted by [27,29,31] but doubted or neglected by [1,10,32].

Although *D. glaciale* subsp. *glaciale* and *D. stiriacum* often co-occur, hybrids have never been reported [29], which is likely caused by the different ploidy levels.

***Doronicum grandiflorum* Lam. (= *D. scorpioides* Lam., *D. halleri* Tausch, *D. portae* Chabert)**

Doronicum grandiflorum is a morphologically variable species of subalpine to subnival calcareous screes, distributed in the Cantabrian Mountains, the Pyrenees, Corsica, the Alps and the Dinarides [25,33,34]. Based on morphological traits such as leaf shape, homomorphic fruits, rhizome and indumentum as well as on its ecology and distribution, *D. grandiflorum* was identified as the closest relative of the *D. clusii* aggregate, placed altogether in subsection *Grandiflora* [27]. The three different hair types present in *D. grandiflorum* – multiseriate glandular trichomes, uni- and multiseriate eglandular trichomes [4,10] – can also be found in members of the *D. clusii* aggregate. Molecular phylogenetic analyses [9] identified *D. grandiflorum* and the Iberian endemic *D. carpetanum* Willk. s.l. as the closest relatives of the *D. clusii* aggregate. Although hybrids of *D. grandiflorum* with *D. clusii* and *D. glaciale* s.s. were never reported [29], hybridization between them cannot be ruled out as they share the chromosome number of $2n = 60$ [35–40] and sometimes co-occur on limestone, or on basic to acidic silicate (C. Pachschwöll, pers. obs.).

References

1. Vierhapper F (1900) „*Arnica Doronicum* Jacquin“ und ihre nächsten Verwandten. Österr Bot Z 50: 109–115, 173–178, 202–208, 257–264, 501.
2. Ehrendorfer F, editor (1973) Liste der Gefäßpflanzen Mitteleuropas, 2nd ed. Stuttgart: Gustav Fischer. 318 p.
3. Fischer MA, editor (1994) Exkursionsflora von Österreich. Stuttgart: Ulmer. 1180 p.
4. Cavillier F (1907) Étude sur les *Doronicum* à fruits homomorphes. Annu Conserv Jard Bot Genève 10: 177–251.
5. Ferguson IK (1976) *Doronicum* L. In: Tutin TG, Heywood VH, Burges NA, Moore DM, Valentine DH, Walters SM, Webb DA, editors. Flora Europaea 4. Cambridge (UK): Cambridge Univ Press. pp. 190–191.

6. Aeschimann D, Lauber K, Moser DM, Theurillat JP (2004) Flora Alpina 2: Gentianaceae–Orchidaceae. Bern: Haupt. 2670 p.
7. Fischer MA, Adler W, Oswald K (2008) Exkursionsflora von Österreich, Liechtenstein und Südtirol. 3rd ed. Linz: Land Oberösterreich, Biologiezentrum der OÖ Landesmuseen. 1391 p.
8. Álvarez Fernández I, Nieto Feliner G (1999) Lectotypification of 16 species names in *Doronicum* (Asteraceae, Senecioneae). Taxon 48: 801–806.
9. Álvarez Fernández I, Fuertes Aguilar J, Panero J, Nieto Feliner G (2001) A phylogenetic analysis of *Doronicum* (Asteraceae, Senecioneae) based on morphological, nuclear ribosomal (ITS), and chloroplast (trnL-F) evidence. Mol Phylogen Evol 20: 41–64.
10. Álvarez Fernández I (2003) Systematics of the Eurasian and North-African genus *Doronicum* (Asteraceae, Senecioneae). Ann Miss Bot Gard 90: 319–389.
11. Merxmüller H (1952) Untersuchungen zur Sippengliederung und Arealbildung in den Alpen. I. Jahrb Vereins Schutze Alpenpfl Alpentiere 17: 96–133.
12. Merxmüller H (1953) Untersuchungen zur Sippengliederung und Arealbildung in den Alpen. II. Jahrb Vereins Schutze Alpenpfl Alpentiere 18: 138–158.
13. Merxmüller H (1954) Untersuchungen zur Sippengliederung und Arealbildung in den Alpen. III. Jahrb Vereins Schutze Alpenpfl Alpentiere 19: 97–139.
14. Magauer M, Schönswetter P, Jang T-S, Frajman B (2014) Disentangling relationships within the disjunctly distributed *Alyssum ovirense*/*A. wulfenianum* group (Brassicaceae), including description of a novel species from the north-eastern Alps. Bot J Linn Soc 176: 486–505.
15. Tribsch A (2004) Areas of endemism of vascular plants in the Eastern Alps in relation to Pleistocene glaciations. J Biogeogr 31: 747–760.
16. Schönswetter P, Stehlik I, Holderegger R, Tribsch A (2005) Molecular evidence for glacial refugia of mountain plants in the European Alps. Mol Ecol 14: 3547–3555.
17. Staudinger M (2009) *Doronicum glaciale* (Wulf.) Nyman subsp. *calcareum* (Vierh.) Hegi. In: Rabitsch W, Essl F, editors. Endemiten – Kostbarkeiten in Österreichs Pflanzen- und Tierwelt. Klagenfurt: Naturwissenschaftlicher Verein für Kärnten. pp. 124–125
18. Stöhr O (2009) *Doronicum glaciale* (Wulf.) Nyman subsp. *glaciale*. In: Rabitsch W, Essl F, editors. Endemiten – Kostbarkeiten in Österreichs Pflanzen- und Tierwelt. Klagenfurt: Naturwissenschaftlicher Verein für Kärnten. pp. 125–127.
19. FloraFaunaSüdtirol (2014) Das Portal zur Verbreitung von Tier- und Pflanzenarten in Südtirol. Naturmuseum Südtirol, Bozen. Database: FloraFaunaSüdtirol. www.florafauna.it. Accessed 21 August 2014
20. Dalla Torre KW, Sarnthein L (1912) Flora der gefürsteten Grafschaft Tirol, des Landes Vorarlberg und des Fürstenthumes Liechtenstein. VI. Band, 3. Teil. Innsbruck: Wagner‘sche Universitätsbuchhandlung. 956 p.

21. Pachschwöll C, Pușcaș M, Schönswetter P (2011) Distribution of *Doronicum clusii* and *D. stiriacum* (Asteraceae) in the Alps and Carpathians. Biologia (Bratislava) 66: 977–987.
22. Dörr E, Lippert W (2004) Flora des Allgäus und seiner Umgebung 2. Eching bei München: IHW-Verlag. 752 p.
23. Reisigl H, Pitschmann H (1958) Obere Grenzen von Flora und Vegetation in der Nivalstufe der zentralen Ötztaler Alpen (Tirol). Vegetatio 8: 93–129.
24. Badanina VA (1997) Pro vydovu samostiinist' *Doronicum stiriacum* Vill. D.T. u flori Ukrayiny [About species' independence of *Doronicum stiriacum* (Vill.) D.T. in the Ukrainian Flora]. Ukr Bot Zh 54: 372–375.
25. Meusel H, Jäger E (1992) Vergleichende Chorologie der zentraleuropäischen Flora 3. Gustav Fischer, Jena. 688 p.
26. Greuter W (2008) Med-checklist: A critical inventory of vascular plants of the circum-mediterranean countries 2: Dicotyledones (Compositae). Genève: Organisation for the Phyto-Taxonomic Investigation of the Mediterranean Area (OPTIMA).
27. Cavillier F (1911) Nouvelles études sur le genre *Doronicum*. Annu Conserv Jard Bot Genève 13–14: 195–368.
28. Hegi G (1928/29) Illustrierte Flora von Mittel-Europa VI.2. Wien: A. Pichler's Witwe & Sohn. 838 p.
29. Widder FJ (1934) Die Bastarde der *Doronicum*-Arten. Mitt Naturwiss Vereins Steiermark 71: 132–146.
30. Wagenitz G, editor (1987) Illustrierte Flora von Mitteleuropa, 2nd ed., VI/4: Compositae II: *Matricaria – Hieracium*. Berlin: Paul Parey. 904 p.
31. Polatschek A, Neuner W (2013) Flora von Nordtirol, Osttirol und Vorarlberg Band 6. Innsbruck: Tiroler Landesmuseum Ferdinandeum. 973 p.
32. Polatschek A (1997) Flora von Nordtirol, Osttirol und Vorarlberg Band 1. Innsbruck: Tiroler Landesmuseum Ferdinandeum. 1024 p.
33. Hayek A (1915) Über einige kritische Pflanzen der Alpenkette. II. *Doronicum Portae* CHAB. Allg Bot Z Syst Floristik Pflanzengeogr 21: 97–102.
34. Frajman B, Pachschwöll C, Schönswetter P (2014) Contributions to the knowledge of the flora of the Dinarides (Balkan Peninsula). Phyton (Horn) 54: 27–46.
35. Favarger C (1949) Notes de caryologie alpine. Bull Soc Neuchâtel Sci Nat 72: 15–42.
36. Favarger C (1950) Polyploïdie et vicariance dans la flore alpine. Arch Julius Klaus-Stift Vererbungsf 25: 472–477.
37. Favarger C (1991) Liens génétiques entre la flore orophile des Tatras et celle des Alpes à la lumière de quelques complexes polyploïdes. Pol Bot Stud 2: 23–38.
38. Favarger C, Küpfer P (1968) Contribution à la cytotaxonomie de la flore alpine des Pyrénées. Collect Bot (Barcelona) 6: 325–352.

39. Lovka M, Sušnik F, Löve Á, Löve D (1972) *Doronicum glaciale* (Wulf.) Nyman. In: Löve Á, editor. IOPB chromosome number reports XXXVI. Taxon 21: 333–346.
40. Huber W, Baltisberger M (1992) IOPB chromosome data 4. Int Organ Plant Biosyst Newslett 18/19: 6–8.
